# Blood flow restriction training for lower-limb function, walking and functional recovery after stroke: a systematic review and meta-analysis

**DOI:** 10.3389/fneur.2026.1987031

**Published:** 2026-10-26

**Authors:** Honghu Kang, Zhexuan Yan, Zhongrui Zhang, Yushan Xu, Wenjing Jiang, Zhongsheng Yang, Qian Zhang, Yu Yin

**Affiliations:** 1 School of Integrated Traditional Chinese and Western Medicine, Hebei University of Chinese Medicine, Shijiazhuang, Hebei, China; 2 Hebei General Hospital, Shijiazhuang, Hebei, China; 3 School of Physical Education, Hebei Normal University, Shijiazhuang, Hebei, China; 4 Graduate School, Hebei Medical University, Shijiazhuang, Hebei, China; 5 School of Nursing and Rehabilitation, North China University of Science and Technology, Tangshan, Hebei, China

**Keywords:** balance, blood flow restriction training, gait, lower-limb function, meta-analysis, muscle strength, rehabilitation, stroke

## Abstract

**Background:**

Blood flow restriction training (BFRT) is increasingly used as a low-load rehabilitation strategy after stroke, but the magnitude, clinical relevance, and certainty of its effects remain uncertain. We evaluated the effects and safety of BFRT on lower-limb function and mobility after stroke.

**Methods:**

PubMed, Embase, Web of Science Core Collection, CENTRAL, CNKI, Wanfang, VIP, and SinoMed were searched from inception to June 10, 2026. Randomized controlled trials evaluating lower-limb BFRT in adults after stroke were included. Risk of bias was assessed using RoB 2, certainty using GRADE, and random-effects meta-analyses were performed.

**Results:**

Nineteen randomized controlled trials were included. BFRT improved Fugl-Meyer Assessment of the Lower Extremity scores (10 studies, 646 participants; MD = 2.14 points, 95% CI 0.74 to 3.54) and Berg Balance Scale scores (8 studies, 581 participants; MD = 4.72 points, 95% CI 1.62 to 7.82). The 10-meter walk test analysis showed higher walking speed (4 studies, 322 participants; MD = 0.14 m/s, 95% CI 0.03 to 0.25), but the estimate attenuated after eligible grey literature was included (6 studies, 410 participants; MD = 0.09 m/s, 95% CI − 0.03 to 0.21). Six-minute walk distance increased by 8.01 m (5 studies, 271 participants; 95% CI 2.31 to 13.71). No clear pooled effects were observed for Timed Up and Go, sit-to-stand performance, cadence, or standard full-scale Barthel Index/Modified Barthel Index scores. FMA-LE and 6MWT effects were below reported clinically important thresholds; the BBS estimate was close to an approximately 5-point reference, with its confidence interval spanning effects below and above that threshold. Certainty was moderate for 6MWT, low for FMA-LE, and very low for BBS and 10MWT walking speed. No BFRT-related serious adverse or thrombotic events were reported among studies with explicit safety information, although reporting was limited.

**Conclusion:**

BFRT may improve selected lower-limb outcomes after stroke, but clinical importance remains uncertain for several outcomes and certainty ranges from moderate to very low. Better-designed trials with standardized BFRT protocols and more complete safety reporting are needed.

**Systematic review registration:**

https://www.crd.york.ac.uk/PROSPERO/view/CRD420261477739, PROSPERO CRD420261477739.

## Introduction

Stroke remains a major cause of long-term disability (1), and persistent lower-limb impairment is a major contributor to reduced mobility and independence after stroke. Weakness, impaired motor control, balance deficits, and reduced walking capacity can limit standing, transfers, community ambulation, and activities of daily living (2). Lower-limb strength is closely related to walking performance after stroke (3), and restoring motor function, balance, and walking ability remains a central goal of rehabilitation. Contemporary stroke rehabilitation therefore emphasizes progressive exercise, repetitive practice, and task-oriented training to improve the functional capacity of the paretic lower limb (2).

Resistance training is an established approach for improving muscle strength after stroke, but conventional higher-load exercise may be difficult for some patients with marked weakness, frailty, poor motor control, or limited exercise tolerance (4). Blood flow restriction training (BFRT) has attracted interest because it can increase the physiological stimulus of exercise while relatively low external loads are used. BFRT typically applies external pressure to the proximal limb, partially restricting arterial inflow and more substantially restricting venous outflow during exercise (5). The resulting metabolic stress and fatigue may increase motor-unit recruitment and promote muscular adaptation despite lower mechanical loading (6). Evidence from exercise and musculoskeletal rehabilitation suggests that low-load exercise combined with blood flow restriction can improve muscle strength and muscle mass, providing a rationale for its use in patients who may not tolerate conventional high-load resistance training (7). BFRT has consequently been introduced into stroke rehabilitation through resistance exercise (8, 9), cycling (10, 11), and other lower-limb training approaches (12), with early randomized trials reporting improvements in muscle performance and selected functional outcomes.

Evidence for BFRT in neurological rehabilitation has developed rapidly. Vinolo-Gil et al. initially reviewed seven studies across several neurological disorders, when the available evidence remained limited and heterogeneous (13). Jønsson et al. subsequently identified a larger body of neurological BFRT research (14), while more recent work by Wang et al. synthesized 37 studies involving 963 participants and reported favorable effects on muscle strength and several functional outcomes (15). Stroke-specific evidence has also become available. Zhang et al. included 11 randomized trials involving 522 participants and examined lower-limb motor function, balance, walking ability, activities of daily living, and the influence of intervention duration (16). These studies have established an important evidence base, but several questions remain incompletely resolved. Existing syntheses have concentrated mainly on a limited set of functional outcomes, while other clinically relevant aspects of lower-limb rehabilitation, including short-distance walking performance, sit-to-stand ability, muscle strength, gait speed, and cadence, have received less integrated evaluation (15, 16). BFRT protocols also differ considerably in exercise modality, cuff application, pressure prescription, training intensity, treatment duration, and comparator design, making it important to consider the clinical and treatment characteristics underlying the pooled effects (17, 18). Zhang et al. also highlighted substantial variation in pressure, cuff placement, training frequency, and other protocol features across the available stroke literature (16). Despite the growing number of trials, the certainty of evidence supporting BFRT after stroke remains unclear, particularly in the context of substantial variation in pressure prescription, cuff characteristics, exercise modality, intervention duration, and comparator design (16). Establishing outcome-specific certainty is therefore important for determining how confidently the observed pooled effects can inform clinical rehabilitation.

Safety is an important consideration when BFRT is applied after stroke because the intervention deliberately alters limb blood flow during exercise through external cuff pressure (5, 6). Its clinical value therefore depends not only on functional efficacy, but also on whether it can be delivered without clinically important adverse effects or treatment intolerance. However, safety has received less consistent evaluation than functional outcomes in the available stroke BFRT literature. Accordingly, the present review assessed reported adverse events, treatment-related withdrawals, and other available safety information alongside functional outcomes.

We therefore conducted a systematic review and meta-analysis of randomized controlled trials evaluating BFRT after stroke. We aimed to investigate its effects on lower-limb motor function, balance, walking performance and endurance, functional mobility, muscle strength, gait characteristics, and activities of daily living. We also summarized key clinical and treatment-related factors, including stroke stage, exercise modality, cuff application, pressure-prescription method, exercise intensity, intervention duration, comparator condition, follow-up, and safety. By examining these rehabilitation domains separately, we sought to identify where the evidence for BFRT is most consistent and to provide a clearer basis for the design and clinical interpretation of future BFRT trials after stroke.

## Methods

### Protocol and reporting

This systematic review and meta-analysis was conducted in accordance with the Cochrane Handbook for Systematic Reviews of Interventions (19) and reported according to the Preferred Reporting Items for Systematic Reviews and Meta-Analyses (PRISMA) guidelines (20). This systematic review was registered with PROSPERO (CRD420261477739). Registration occurred after initiation of the review and is reported transparently. As this review was based exclusively on aggregate data from previously published studies, ethical approval was not required.

### Search strategy and data sources

A systematic literature search was conducted in PubMed, Embase, Web of Science Core Collection, the Cochrane Central Register of Controlled Trials (CENTRAL), China National Knowledge Infrastructure (CNKI), Wanfang Data, VIP Database, and SinoMed/Chinese Biomedical Literature Database (CBM), from database inception to June 10, 2026. The search strategy was developed around two core concepts: stroke and blood flow restriction training. Intervention-related terms included blood flow restriction, blood flow restriction training, occlusion training, KAATSU, BFR, BFRT, BFRE, and related expressions. Controlled vocabulary and free-text terms were combined where appropriate, and the search syntax was adapted to the requirements of each database.

To maximize search sensitivity, lower-extremity terms, specific functional outcomes, and randomized controlled trial filters were not used as mandatory search components. Eligibility criteria were instead applied during study screening. No language restrictions were imposed during the literature search or study-selection process. In addition, the reference lists of included studies and relevant systematic reviews were manually screened to identify further potentially eligible studies. The complete search strategies for all databases are provided in Supplementary Table S1.

### Eligibility criteria

Studies were considered eligible according to the following PICOS criteria.

Participants: Eligible participants were adults aged 18 years or older with a clinical diagnosis of ischemic or hemorrhagic stroke. Studies were eligible regardless of sex, stroke stage, or time since stroke, provided that the diagnosis was clearly established. Stroke stage and other clinical characteristics were not used as eligibility restrictions and were instead considered potential sources of clinical heterogeneity.

Interventions: Eligible interventions involved blood flow restriction training (BFRT) applied during lower-limb exercise or rehabilitation. BFRT could be delivered alone or in combination with structured rehabilitation and could include resistance exercise, cycling or aerobic training, suspension-based exercise, whole-body vibration, functional exercise, or other lower-limb rehabilitation approaches. External restriction could be applied using pneumatic cuffs, tourniquet systems, pressure bands, or comparable devices. No restrictions were imposed on cuff type, cuff width, pressure-prescription method, target pressure, or the use of individualized arterial occlusion pressure, as these characteristics were considered potential sources of intervention-level heterogeneity.

Comparators: Eligible comparators included the same or a comparable exercise or rehabilitation program performed without meaningful blood flow restriction, sham or minimal-pressure restriction, another active exercise intervention, or routine rehabilitation. For multi-arm trials, the comparison that most directly isolated the additional effect of blood flow restriction was prioritized. When multiple eligible BFRT arms shared a common control group, intervention arms were combined where appropriate to avoid double counting of the shared comparator.

Outcomes: Studies were required to report at least one outcome related to lower-limb motor function, balance, walking performance or endurance, functional mobility, lower-limb muscle strength, gait characteristics, activities of daily living, or safety. The principal functional outcomes included lower-limb motor impairment assessed using the Fugl-Meyer Assessment of the Lower Extremity (FMA-LE) and balance assessed using the Berg Balance Scale (BBS). Additional outcomes included walking performance assessed using the 10-meter walk test (10MWT), walking endurance assessed using the 6-min walk test (6MWT), functional mobility assessed using the Timed Up and Go test (TUG) and sit-to-stand tests, lower-limb muscle strength such as knee extensor peak torque, gait characteristics including gait speed and cadence, and activities of daily living assessed using the Barthel Index (BI), Modified Barthel Index (MBI), or clinically comparable measures. Safety outcomes, including adverse events and treatment-related withdrawals, were also extracted where reported.

Study design and publication type: Only randomized controlled trials reported as full-text journal articles were eligible. Reviews, systematic reviews, protocols, observational or other non-randomized studies, case reports, animal studies, duplicate publications, reports of overlapping samples without independent data, master’s or doctoral theses/dissertations, conference abstracts or proceedings, registry-only records, and other reports without sufficient extractable primary trial data were excluded.

The primary synthesis was restricted to randomized controlled trials reported as full-text journal articles because the review required sufficient methodological, intervention, and outcome detail for reproducible eligibility assessment, data extraction, outcome harmonization, and outcome-level risk-of-bias evaluation. We acknowledge that excluding grey literature may omit unpublished or non-journal evidence and could contribute to publication-related bias. To examine the robustness of the primary findings to this eligibility decision, potentially eligible grey-literature trials identified during the search were retained and incorporated into additional sensitivity analyses when sufficient outcome data were available.

### Study selection

All records identified through the database searches were imported into reference management software, and duplicate records were removed before screening. Two reviewers independently screened the titles and abstracts of the remaining records to identify potentially eligible studies. Full-text articles were then retrieved and assessed independently against the predefined eligibility criteria. Any disagreements were resolved through discussion, with a third reviewer consulted when consensus could not be reached. Reasons for exclusion at the full-text stage were documented, and the overall study-selection process was presented in a PRISMA flow diagram.

### Data extraction

Two reviewers independently extracted data from the included studies using a standardized data extraction form. Extracted information included study characteristics; participant characteristics such as sample size, age, sex, stroke type, stroke stage, and time since stroke; BFRT and comparator interventions; concomitant rehabilitation; outcome measures; follow-up; and adverse events. Detailed BFRT characteristics were also extracted, including cuff or device type, cuff width and placement, pressure-prescription method, target pressure, arterial or limb occlusion pressure determination where applicable, pressurization and deflation pattern, exercise modality and intensity, session duration, training frequency, and total intervention duration.

For continuous outcomes, group-specific sample sizes, means, and standard deviations were extracted whenever available. Numerical data presented graphically were used when they could be extracted with sufficient reliability, and the influence of figure-derived data was examined in sensitivity analyses where appropriate. Outcomes for which sufficiently compatible quantitative data could not be obtained were retained for descriptive or narrative synthesis.

### Outcome definition and data harmonization

Outcomes were grouped according to clinically comparable constructs before quantitative synthesis. Only lower-extremity Fugl-Meyer outcomes were included in the FMA-LE analysis, whereas total FMA scores were not treated as equivalent to the lower-extremity subscale. Standard full BI or MBI scores were analyzed separately from non-standard lower-limb ADL subscores. Completion-time sit-to-stand tests were distinguished from repetition-based sit-to-stand measures, and gait-speed data were converted to a common unit where necessary. For time-based outcomes, effect directions were aligned so that positive values consistently indicated improvement in favor of BFRT.

For studies reporting the 10MWT as completion time, group-level walking speed was approximated in meters per second by dividing the actual timed distance by the reported mean completion time. The corresponding standard deviation was approximated using the first-order delta method (SDspeed ≈ distance × SDtime / mean time^2^). The actual timed segment reported by each study was used. In multi-arm trials, eligible BFRT arms were transformed separately before being combined using standard formulas, with the shared control group included only once. For studies reporting repeated assessments, the primary pooled analyses used post-intervention group values from the end-of-treatment assessment. Accordingly, MDs and SMDs represented between-group differences in post-intervention values rather than differences in change-from-baseline scores, unless otherwise specified. Additional data-harmonization details are provided in Supplementary Table S2.

### Risk of bias assessment

Risk of bias was assessed using the Cochrane Risk of Bias 2 (RoB 2) tool (21) at the level of specific outcome results. Outcome-specific assessments were conducted for study results contributing to the principal syntheses of FMA-LE, BBS, 10MWT, 6MWT, and standard full-scale BI/MBI. The five RoB 2 domains comprised bias arising from the randomization process, bias due to deviations from intended interventions, bias due to missing outcome data, bias in measurement of the outcome, and bias in selection of the reported result. Where the same trial contributed to more than one principal outcome, a separate set of judgments was recorded for each relevant outcome result. Overall judgments were categorized as low risk, some concerns, or high risk. Detailed outcome-specific judgments are provided in Supplementary Table S3.

Two reviewers independently completed the RoB 2 assessments. Any disagreements were first discussed between the two reviewers, and unresolved discrepancies were adjudicated by a third reviewer.

### Certainty of evidence

The certainty of evidence for each major outcome was evaluated using the Grading of Recommendations Assessment, Development and Evaluation (GRADE) approach (22). Evidence was assessed across five domains: risk of bias, inconsistency, indirectness, imprecision, and publication bias. The overall certainty of evidence was classified as high, moderate, low, or very low.

Two reviewers independently completed the GRADE assessments for each major outcome. Disagreements regarding individual domains or overall certainty ratings were discussed, and any unresolved discrepancies were adjudicated by a third reviewer.

### Data synthesis and statistical analysis

Meta-analysis was performed when at least two studies provided clinically and methodologically comparable data for the same outcome. Continuous outcomes were expressed as mean differences (MDs) with 95% confidence intervals when the same or directly comparable scales were used, whereas standardized mean differences (SMDs) were calculated when conceptually similar outcomes were assessed using different scales. For time-based outcomes, effect directions were harmonized so that positive values consistently indicated improvement in favor of BFRT.

Random-effects models were used for all pooled analyses because clinical and methodological heterogeneity was anticipated across trials. Potential sources of heterogeneity included stroke characteristics, BFRT training modality, cuff and pressure prescription, exercise intensity and protocol, intervention duration, comparator type, and concomitant rehabilitation. Between-study variance was estimated using restricted maximum likelihood, and Hartung-Knapp adjustment was used for confidence intervals and statistical inference of pooled random-effects estimates because several outcomes included only a small number of studies. Conventional random-effects confidence intervals were retained as sensitivity analyses. Statistical heterogeneity was quantified using the I^2^ statistic, with values of approximately 25%, 50%, and 75% considered to indicate low, moderate, and substantial heterogeneity, respectively. All analyses were performed using R software. All statistical tests were two-sided, and *p* < 0.05 was considered the conventional threshold for statistical significance. No formal adjustment for multiple comparisons was applied. Accordingly, secondary, sensitivity, and exploratory subgroup analyses were interpreted cautiously.

Sensitivity analyses were undertaken to evaluate the robustness of the pooled estimates and the influence of individual studies. Leave-one-out analyses were performed when sufficient studies were available, and additional sensitivity analyses were conducted when concerns arose regarding influential observations, outcome validity, figure-derived data, or alternative outcome definitions. Exploratory subgroup analyses were conducted according to BFRT training modality, comparator type, and intervention duration. Training modality was categorized according to the predominant exercise component as resistance-based, cycling/aerobic, suspension-based, or other/multimodal. Where sparse subgroup cells limited meaningful comparison, conceptually similar non-resistance modalities were combined. Comparator type was classified as a matched active comparator when the control group received the same or a closely comparable exercise or rehabilitation program without meaningful blood flow restriction, and as a routine-rehabilitation control when the control group received routine rehabilitation without a specifically matched exercise component. Intervention duration was categorized as ≤4 weeks or >4 weeks. Subgroup analyses were not retained when any category contained only a single study. Small-study effects were assessed only when at least 10 studies contributed to an outcome, using funnel plot inspection, Egger regression, the Begg-Mazumdar rank correlation test, and trim-and-fill analysis where appropriate.

## Results

### Study selection

A total of 589 records were identified from the eight databases. Before screening, 63 degree-thesis or other grey-literature records were removed according to the predefined publication-type eligibility criteria, and 254 duplicate records were removed, leaving 272 records for title and abstract screening. Twenty-nine reports were subsequently assessed in full text, of which 10 were excluded because of ineligible publication type (nine master’s theses/dissertations and one conference proceeding). The 10 reports excluded after full-text assessment and the corresponding reasons for exclusion are listed in Supplementary Table S4. Ultimately, 19 randomized controlled trials were included in the systematic review and meta-analysis, and all contributed data to at least one quantitative synthesis (Figure 1).

**Figure 1:**
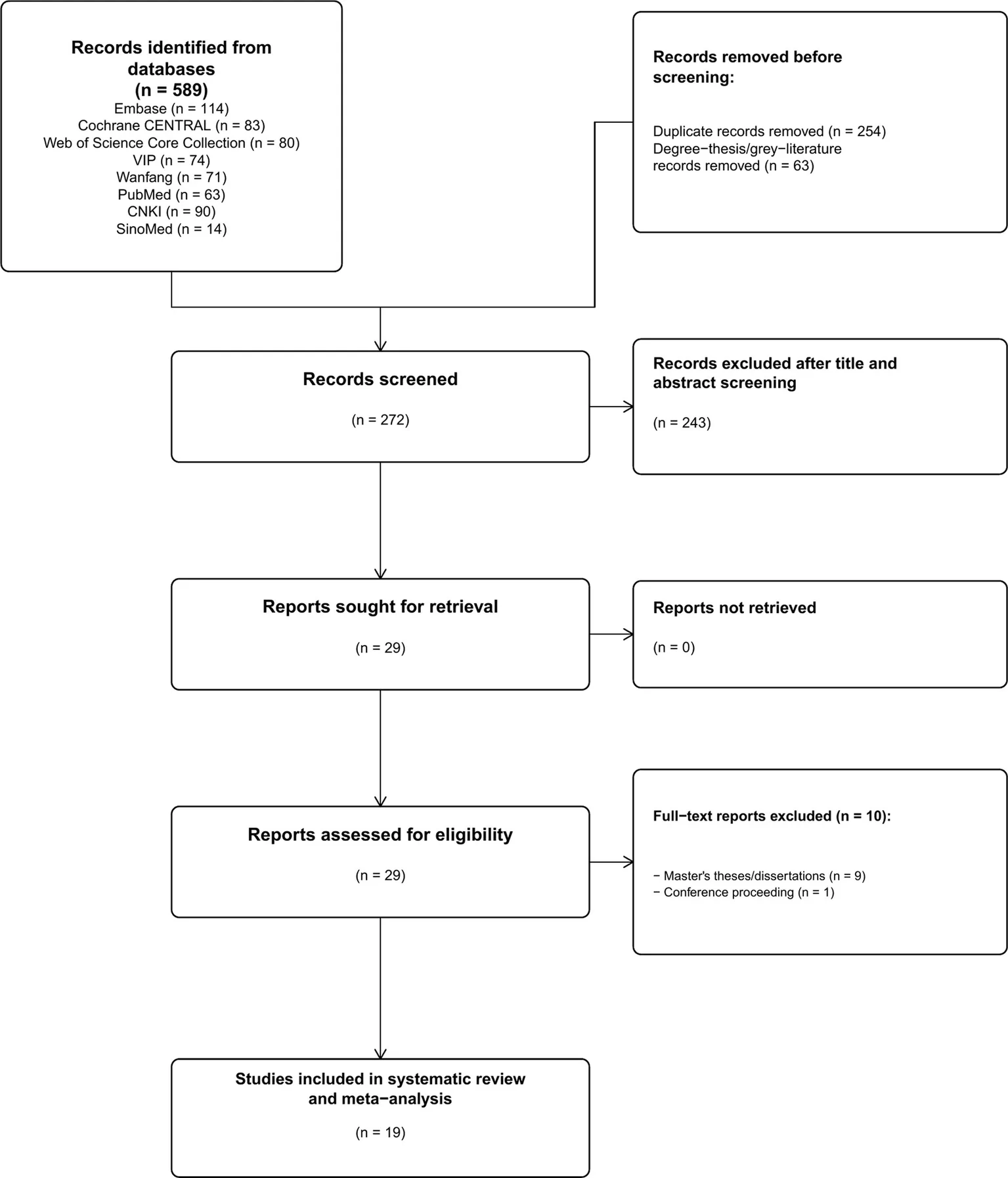
PRISMA flow diagram of the study selection process.

### Characteristics of included studies

The included trials evaluated BFRT in adults after stroke. Most were small randomized trials, and BFRT was commonly delivered alongside rehabilitation-based exercise, including low-load resistance training, cycling or aerobic exercise, suspension training, whole-body vibration, functional exercise, or routine rehabilitation. Eighteen studies were conducted in China and one in Turkey. The study populations included patients with ischemic or hemorrhagic stroke across different stages of recovery. The main characteristics of the included studies are summarized in Table 1, with additional participant information provided in Supplementary Tables S5A,B.

**Table 1:** Characteristics of the included studies.

Study characteristics		Intervention characteristics				Outcomes
Study	Participants	Experimental group	Comparator	BFR parameters	Treatment dose	
Qin et al. (12) China	*N* = 80 analyzed (40/40)^a^ Ischemic or hemorrhagic stroke with hemiplegia Stroke timing: 45.7 ± 14.6 d vs. 45.9 ± 13.9 d	Routine rehabilitation + BFRT with structured low-intensity active lower-limb exercise (≈20–30% estimated 1RM)	Routine rehabilitation	160–170 mmHg; 7-cm pneumatic cuff; proximal affected thigh; two pressurized cycles with release between cycles	≈20 min/day; 5 d/wk.; 8 wk	FMA-LE; BBS; 6MWT; MBI; lower-limb MMT
Bai et al. (10) China	*N* = 66 randomized; 60 analyzed (20/group) First subcortical ischemic stroke with hemiparesis 2 wk–6 mo post-stroke	30% 1RM MOTOmed resistance cycling + BFR	Matched low-load cycling (30% 1RM); high-load cycling (80% 1RM) retained as a separate comparator stratum	200 mmHg; 7-cm B STRONG cuff; inguinal crease/proximal affected limb; 3 min pressurized + 1 min release ×5	20 min/session; 5 sessions/wk.; 4 wk	Rectus femoris CSA; knee extensor PT; FMA-LE; fNIRS cortical activation
Li et al. (11) China	*N* = 34 (17/17) Stroke patients able to perform cycle-ergometer training < 6 mo post-stroke	Cycle ergometer training + BFR	Same cycle ergometer training + 10% AOP sham-like pressure	40–80% AOP; 10-cm cuff at proximal thigh; 2 min exercise + 1 min rest	2 sessions/day; 5 d/wk.; 3 wk	Rectus femoris thickness/CSA; gastrocnemius pennation angle; 30-s STS; FMA-LE
Feng et al. (9) China	*N* = 40 randomized; 38 post-treatment (20/18) Hemiparetic ischemic or hemorrhagic stroke Median time since stroke: 7.0 mo (IQR 4.0–23.5) in BFRET and 6.0 mo (IQR 3.5–24.0) in ET.	Blood-flow-restricted exercise training (BFRET)	Exercise training (ET), 30 min twice/day; total exercise dose differed between groups	160–200 mmHg; B STRONG bands at proximal thigh; pressure maintained and monitored during BFRET	30 min BFRET/day; 20 sessions over 4 wk	MMT; FMA-LE; TUG; rectus femoris/vastus lateralis/tibialis anterior muscle thickness
Ahmed et al. (8) Turkey	*N* = 32 randomized; 30 analyzed (15/15) Ischemic stroke survivors; functionally ambulatory Mean time since stroke ≈24–26 mo	Low-intensity resistance training + BFR (40% 1RM)	High-intensity resistance training (80% 1RM)	150–160 mmHg; proximal affected lower limb; 3 sets	≈3 sessions/wk.; 5 wk	6MWT; five-time Sit-to-Stand Test (5TSTST); TUG; BI; gait speed; stride length; cadence; HADS
An et al. (44) China	*N* = 200 randomized; 196 analyzed (49/group) Older adults with ischemic stroke and frailty Stroke timing: 15.10 ± 1.16 d, 15.30 ± 1.12 d, 15.60 ± 1.12 d, and 15.28 ± 0.94 d	30% 1RM resistance + BFRT at 40%, 50%, or 60% AOP	30% 1RM resistance training	AOP-based; 40%, 50%, or 60% AOP; lower-limb cuff near groin crease	≈15 min/session; 2 sessions/wk.; 8 wk	FMA-LE; BBS; 10MWT; FMA-UE; grip strength; FFP; MAS
Sun et al. (45) China	*N* = 80 (WBV + iBFR 27; WBV 28; routine 25) Older convalescent stroke patients with hemiplegia Mean disease duration ≈43–52 d	Whole-body vibration (WBV) + intermittent blood flow restriction (iBFR)	WBV alone as the primary incremental comparator; routine rehabilitation as third arm	≤1.3 × SBP (≈140–200 mmHg); proximal thigh; 3 min inflation + 1 min release	≈15 min component; 5 d/wk.; 6 wk	BBS; 6MWT; CB&M; 1- and 3-mo follow-up
Luo et al. (23) China	*N* = 40 (20/20) Post-stroke lower-limb dysfunction Stroke timing: NR	Routine rehabilitation + exercise therapy + pressure/BFR training	Routine rehabilitation + exercise therapy	0.01–0.03 MPa; proximal lower limb; pressure applied during power-cycle training	10–20 min/day; ≈3 wk	Lower-limb FMA; MMT (FMA-LE excluded from the primary synthesis because the reported mean exceeded the scale maximum; retained in sensitivity analysis.)
Gong et al. (46) China	*N* = 52 (26/26) Stroke patients receiving rehabilitation Stroke timing: 26.19 ± 4.60 d vs. 27.42 ± 6.73 d	20% 1RM MOTOmed cycling + scalp acupuncture + BFR	20% 1RM MOTOmed cycling + scalp acupuncture	120 mmHg; 10 × 70 cm cuff at proximal affected thigh; 3 min inflation + 1 min relaxation ×5	20 min/session; 2 sessions/day; 4 wk	BBS; MMT; instrumented balance; cycling resistance; thigh circumference
Chen et al. (41) China	*N* = 71 (35/36) Older patients with cerebral infarction and sarcopenia Early post-stroke; exact stage NR	Low-intensity resistance training + BFRT	Low-intensity resistance training	≤1.3 × SBP (≈140–200 mmHg); cuff applied proximally	12 wk	MBI; ASM; SMI; ASMI; SPPB; grip strength; GH; IGF-1; IL-6
Xu et al. (40) China	*N* = 58 (29/29) Stroke inpatients with unilateral paralysis; Brunnstrom III–IV Stable; ≤6 mo post-stroke	Routine rehabilitation + 20% 1RM MOTOmed cycling + BFR	Routine rehabilitation + 20% 1RM MOTOmed cycling	200 mmHg; thigh cuff ≈10 cm above patella; 3 min inflation + 1 min release ×5	20 min/session; 2 sessions/day; 6 d/wk.; 2 wk	FMA-LE; knee extensor/flexor PT and PT/W; 1RM; sEMG
Shang et al. (47) China	*N* = 40 (20/20) Stroke patients with lower-limb dysfunction Stroke timing: 8.20 ± 1.82 wk. vs. 8.67 ± 1.72 wk	Routine rehabilitation + suspension training + BFR	Routine rehabilitation + suspension training	200 mmHg; thigh cuff ≈10 cm above patella; 5 min inflation + 1 min release ×5	1 session/day; 5 d/wk.; 4 wk	10MWT; FMA-LE; MBI; gait parameters; instrumented balance
Guo et al. (48) China	*N* = 40 (20/20) Stroke with genu recurvatum/knee hyperextension Stroke timing: 11.65 ± 4.34 wk. vs. 12.00 ± 3.92 wk	Routine rehabilitation + Redcord suspension training + BFR	Routine rehabilitation + suspension training	200 mmHg; proximal thigh; 5 min inflation + 1 min release ×5	1 session/day; 4 wk	Knee hyperextension measures; BBS; 10MWT; FMA-LE; MBI
Du Yan et al. (49) China	*N* = 60 (30/30) Older stroke patients with hemiplegia Stroke timing: 76.4 ± 18.4 d vs. 75.6 ± 11.1 d	Low-load lower-limb resistance training + BFR	Same lower-limb resistance training	10–30% 1RM; ≤1.3 × SBP (140–200 mmHg); 7 × 50 cm cuff at affected mid-thigh; inflated before each set and released between sets	30 min/day; 5 d/wk.; 8 wk	Lower-limb MMT; FMA-LE; BBS; MBI
Ji et al. (50) China	*N* = 46 (23/23) First-onset unilateral stroke; able to stand and walk 1–3 mo post-stroke	Aerobic power-cycling + BFR	Aerobic power-cycling	160 mmHg; 10-cm cuff at proximal affected thigh; 10 min training + 5 min release/rest + 10 min training	20 min/session; 5 sessions/wk.; 8 wk	10MWT; 6MWT; FTSTS (five-times sit-to-stand test); balance-related measures
Feng et al. (51) China	*N* = 34 randomized; 29 completed (15 BFR-ET/14 ET) Stroke with hemiplegic lower-limb dysfunction Stroke timing: 12.27 ± 11.55 mo vs. 14.36 ± 15.45 mo	BFR-ET + exercise training	Exercise training twice daily	160–200 mmHg; B STRONG bands at proximal thigh; pressure monitored during training	Experimental: 20 min BFR-ET + 30 min ET/day; control: 30 min ET twice/day; 20 d	Brunnstrom stage; MMT; MAS; AROM; FMA-LE; TUG; MBI
Tang et al. (52) China	*N* = 32 (16/16) First unilateral basal-ganglia ischemic stroke Within 8 wk. of onset	Routine rehabilitation + BFR squat and straight-leg-raise training	Routine rehabilitation	70 mmHg; proximal thigh near inguinal region; 30–15–15-15 repetition scheme	5 d/wk.; 2 wk	BBS; instrumented balance/proprioception
Yang et al. (53) China	*N* = 66 (33/33) Obese stroke patients with hemiplegia ≈ 11–25 d post-stroke	Resistance training + BFR	Resistance training	≈20% maximal voluntary contraction; thigh 80–100 mmHg and mid-femur 160–170 mmHg; 7 × 50 cm pressure band	1–2 sessions/day; 5 d/wk.; 6 wk	MMT/strength grades; BBS; total FMA (0–100; not pooled with FMA-LE)
Du Jinnan et al. (54) China	*N* = 60 (30/30) Stroke patients receiving rehabilitation Stroke timing: NR	Routine rehabilitation + 20-Hz rTMS + BFR	Routine rehabilitation + 20-Hz rTMS	250 mmHg; B STRONG band at affected mid-femur; 10 min inflation + 1 min rest + 10 min inflation; adjunct/sequential delivery	1 session/day; 5 d/wk.; 6 wk	FMA-LE; MBI-derived lower-limb ADL subscore (4 items; maximum 55 points); lower-limb strength; 6MWT

1RM, one-repetition maximum; 5TSTST, five-time Sit-to-Stand Test; 6MWT, 6-Minute Walk Test; 10MWT, 10-Meter Walk Test; 30-s STS, 30-s sit-to-stand test; AOP, arterial occlusion pressure; AROM, active range of motion; ASM, appendicular skeletal muscle mass; ASMI, appendicular skeletal muscle mass index; BBS, Berg Balance Scale; BFR, blood flow restriction; BFR-ET, blood flow restriction combined with exercise training; BFRET, blood flow-restricted exercise training; BFRT, blood flow restriction training; BI, Barthel Index; CB&M, Community Balance and Mobility Scale; CSA, cross-sectional area; ET, exercise training; FFP, Fried Frailty Phenotype; FMA, Fugl-Meyer Assessment; FMA-LE, Fugl-Meyer Assessment of the Lower Extremity; FMA-UE, Fugl-Meyer Assessment of the Upper Extremity; fNIRS, functional near-infrared spectroscopy; FTSTS, five-times sit-to-stand test; GH, growth hormone; HADS, Hospital Anxiety and Depression Scale; iBFR, intermittent blood flow restriction; IGF-1, insulin-like growth factor-1; IL-6, interleukin-6; IQR, interquartile range; MAS, modified Ashworth Scale; MBI, Modified Barthel Index; MMT, manual muscle testing; NR, not reported; PT, peak torque; PT/W, peak torque/weight; rTMS, repetitive transcranial magnetic stimulation; SBP, systolic blood pressure; sEMG, surface electromyography; SMI, skeletal muscle index; SPPB, short physical performance battery; TUG, Timed Up and Go; WBV, whole-body vibration.

Multi-arm comparator handling and endpoint-specific data-harmonization decisions are detailed in the Methods and Supplementary Table S2.

a Qin et al. (12) sample-size note: The main text reports 82 participants recruited; after randomization, one participant in each group dropped out, leaving 80 participants who completed the study and were included in the analysis. The abstract reports 80 participants (40/40).

BFRT protocols varied across studies. Cuffs were most commonly applied to the proximal thigh or affected lower limb, although several studies also placed restrictive devices on the upper arm when upper-limb exercise was included. Several trials used fixed absolute pressures, with reported targets ranging from 70 to 250 mmHg, whereas others prescribed pressure according to arterial occlusion pressure (AOP) or systolic blood pressure. AOP-based protocols commonly used 40–80% AOP. Cuff width, pressurization pattern, exercise intensity, session frequency, and total treatment duration also differed across trials. Intervention duration ranged from approximately 2 to 12 weeks. Detailed cuff characteristics, pressure prescriptions, and exercise protocols are provided in Supplementary Tables S6A,B.

Control conditions also varied. Most trials compared BFRT plus exercise with the same or a comparable exercise program without meaningful blood flow restriction, whereas several used routine rehabilitation alone as the control condition. Co-interventions included conventional rehabilitation, physiotherapy, occupational therapy, acupuncture, electrical stimulation, and other routine stroke rehabilitation treatments. Longer-term follow-up was uncommon, and most studies reported outcomes at the end of the intervention period. The pooled estimates therefore mainly represent end-of-treatment effects.

Outcomes covered lower-limb motor function, balance, walking performance, functional mobility, muscle strength, gait characteristics, and activities of daily living. FMA-LE, BBS, 10MWT, 6MWT, TUG, sit-to-stand tests, knee extensor peak torque, cadence, gait speed, and BI/MBI were analyzed separately according to their respective clinical constructs. Study-level contributions to the quantitative syntheses are provided in Supplementary Table S7.

### Risk of bias

Across the 32 outcome-specific results contributing to the five principal outcome syntheses, 6 were judged to be at low overall risk of bias and 26 were judged to have some concerns; none was judged to be at high overall risk. Concerns arose most frequently from the randomization process, largely because allocation concealment was incompletely reported in several trials, and from outcome measurement in studies in which assessor blinding was not clearly described for clinician-rated outcomes. Deviations from intended interventions and missing outcome data were generally judged to be at low risk, while concerns regarding selection of the reported result were uncommon. The outcome-specific judgments are presented in Figure 2 and Supplementary Table S3. Detailed GRADE assessments are provided in Supplementary Table S8.

**Figure 2:**
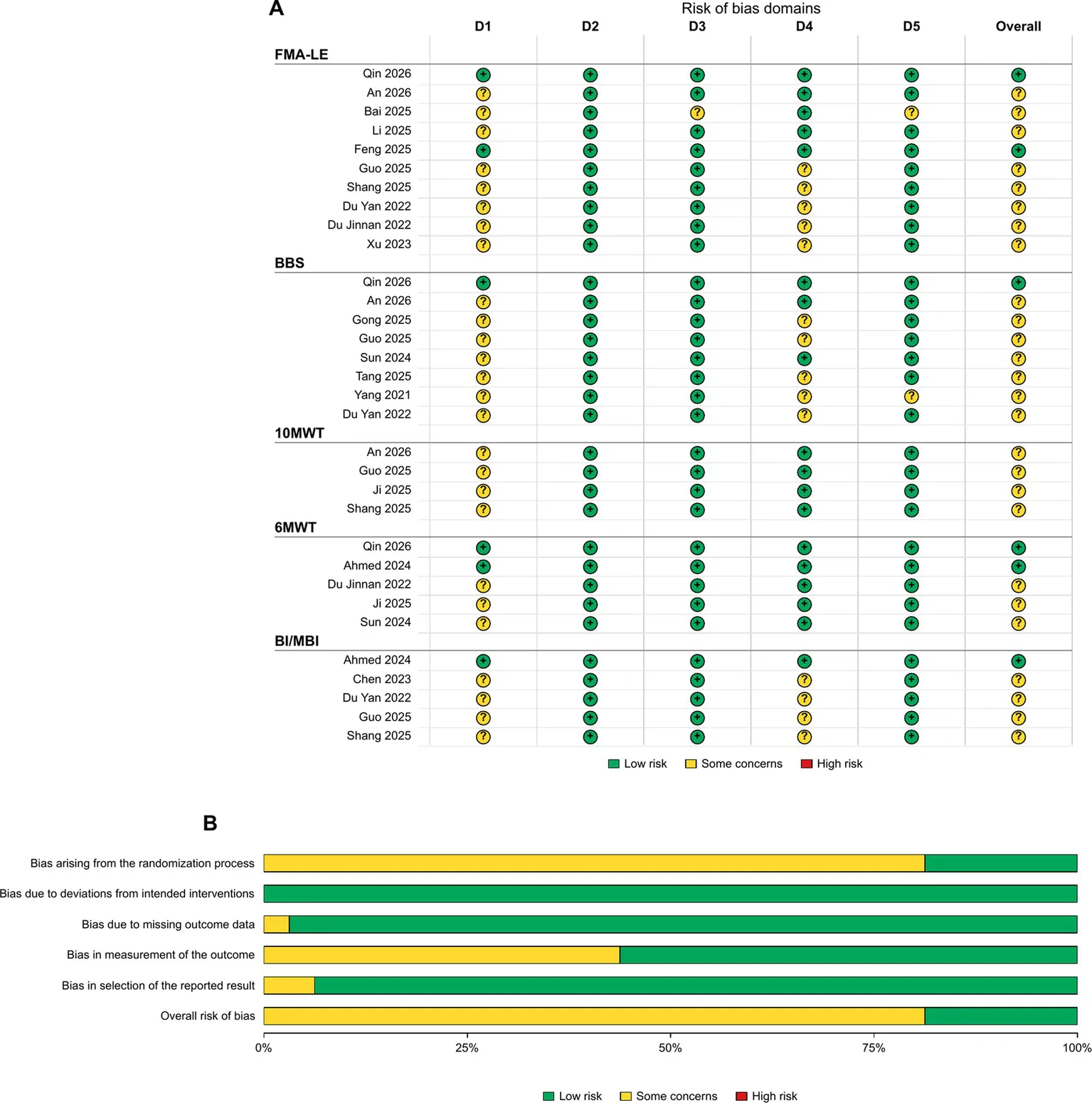
Outcome-specific risk-of-bias assessment using the Cochrane Risk of Bias 2 tool. **(A)** Domain-level judgments for study results contributing to the principal FMA-LE, BBS, 10MWT, 6MWT, and standard full-scale BI/MBI analyses. **(B)** Distribution of risk-of-bias judgments across the outcome-specific results. D1, bias arising from the randomization process; D2, bias due to deviations from intended interventions; D3, bias due to missing outcome data; D4, bias in measurement of the outcome; D5, bias in selection of the reported result.

### Effects on lower-limb motor function: FMA-LE

Ten studies involving 646 participants were included in the primary FMA-LE analysis. Compared with control interventions, BFRT improved lower-limb motor function at the end of treatment, with an average between-group difference of 2.14 points on the 34-point FMA-LE scale (95% CI: 0.74 to 3.54, *p* = 0.007; I^2^ = 72.9%; Figure 3).

**Figure 3:**
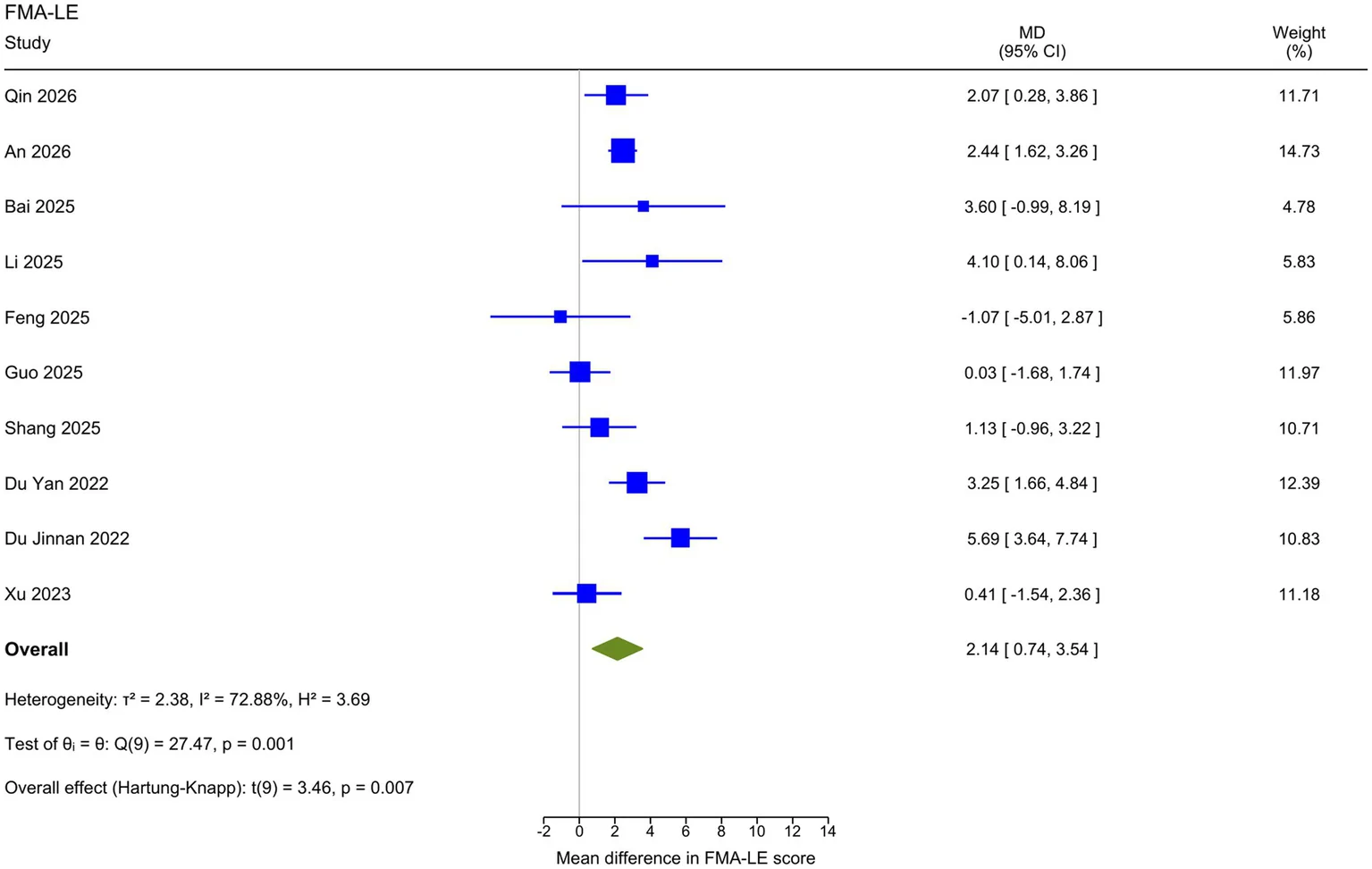
Forest plot of Fugl-Meyer assessment of the lower extremity scores.

The primary analysis excluded the FMA-LE endpoint from Luo et al. (23) because the reported post-treatment mean exceeded the standard scale maximum. Reintroducing this study increased the pooled estimate to 3.31 points (95% CI: 0.77 to 5.86, *p* = 0.011) and markedly increased heterogeneity (I^2^ = 94.9%). In a further sensitivity analysis excluding the figure-digitized FMA-LE data from Li et al. (11), the pooled effect remained significant and close to the primary estimate (9 studies; MD = 2.02 points, 95% CI: 0.76 to 3.28, *p* = 0.002; I^2^ = 75.5%). The FMA-LE finding therefore remained positive across the principal sensitivity analyses, whereas inclusion of Luo et al. (23) primarily affected the magnitude of the effect and between-study heterogeneity. Related sensitivity and influence analyses are shown in Supplementary Figures S1–S4.

Intervention duration showed the clearest pattern in the exploratory subgroup analyses. In six studies with treatment lasting 4 weeks or less, the pooled difference was 0.73 points (95% CI: −0.26 to 1.73; I^2^ = 0%), whereas four studies with treatment lasting more than 4 weeks showed a larger improvement of 3.22 points (95% CI: 1.81 to 4.64; I^2^ = 72.2%). An exploratory between-subgroup difference was observed (*p* = 0.011); however, this analysis was not adjusted for multiple comparisons and should be interpreted cautiously. No significant subgroup difference was observed according to BFRT training modality (*p* = 0.577). Corresponding subgroup analyses are shown in Supplementary Figures S5–S6.

Because 10 studies contributed to the primary FMA-LE analysis, formal small-study-effect analyses were performed. Neither classical Egger regression nor the Begg-Mazumdar test indicated significant small-study effects (*p* = 0.841 and *p* = 1.000, respectively). The mixed-effects Egger analysis yielded a similar result (*p* = 0.962), and trim-and-fill analysis did not materially alter the pooled estimate. Small-study-effect results are provided in Supplementary Table S9 and Supplementary Figures S7, S8, with additional sensitivity and subgroup results summarized in Supplementary Table S10.

### Effects on balance: BBS

Eight studies involving 581 participants reported balance function using the Berg Balance Scale. Compared with control interventions, BFRT produced a 4.72-point greater improvement in BBS score at the end of treatment (95% CI: 1.62 to 7.82, *p* = 0.009; I^2^ = 89.82%, τ^2^ = 11.06, H^2^ = 9.82; Figure 4).

**Figure 4:**
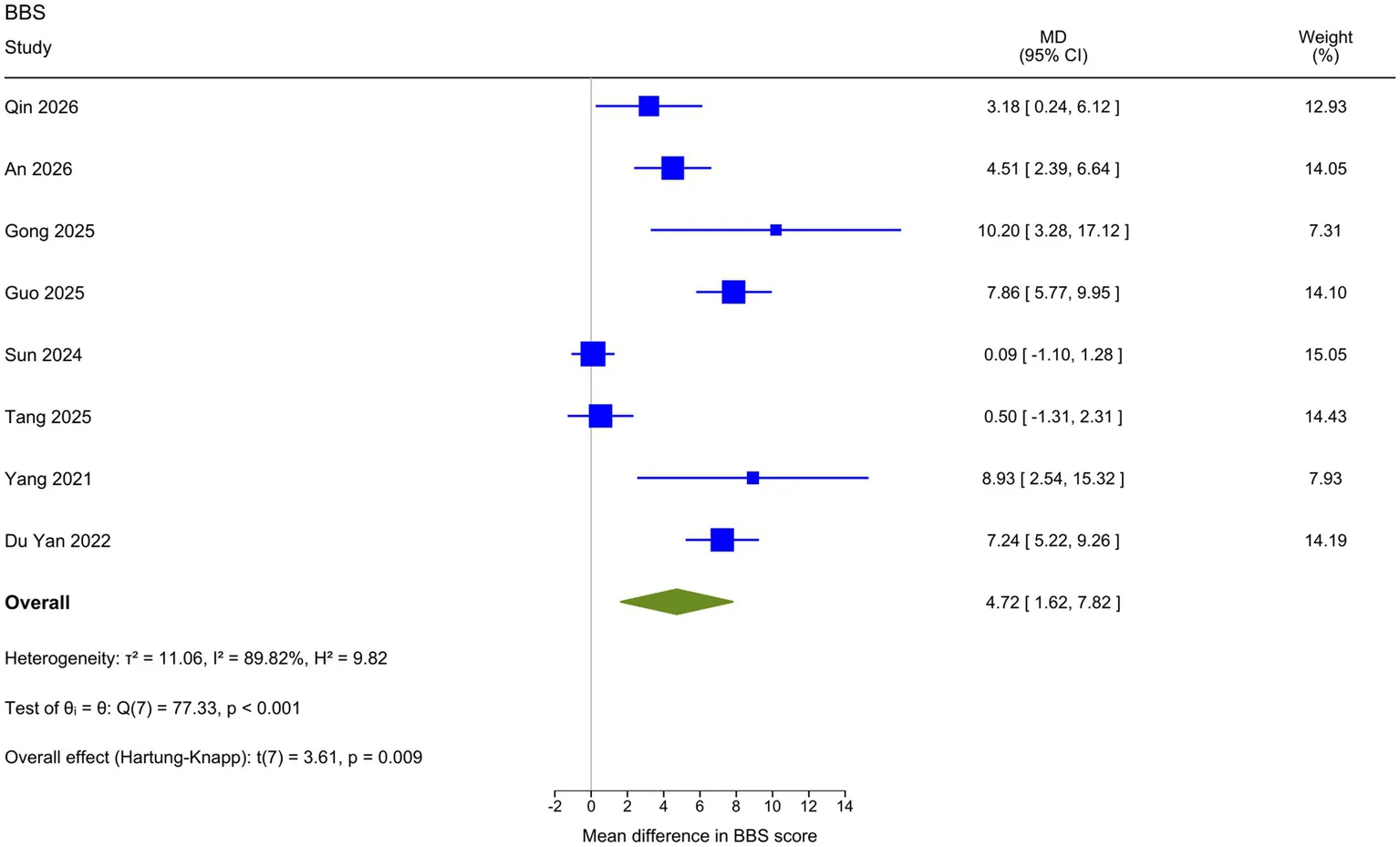
Forest plot of Berg Balance Scale scores.

Leave-one-out sensitivity analyses yielded pooled BBS estimates ranging from 4.18 to 5.46 points, with all corresponding 95% confidence intervals remaining above the null. Heterogeneity remained substantial across all analyses (I^2^ = 83.5–91.7%), indicating that no single study accounted for the observed between-study variability. The leave-one-out analysis is shown in Supplementary Figure S9.

No statistically significant subgroup differences in BBS were detected according to BFRT training modality (*p* = 0.279), comparator type (*p* = 0.149), or intervention duration (*p* = 0.720). Corresponding subgroup analyses are shown in Supplementary Figures S10–S12.

### Effects on walking and mobility

Walking and mobility were evaluated using the 10-meter walk test (10MWT), 6-min walk test (6MWT), Timed Up and Go test (TUG), and sit-to-stand tests.

Four studies involving 322 participants contributed to the 10MWT walking-speed analysis. After conversion of completion-time data to walking speed, the pooled between-group difference favored BFRT (MD = 0.14 m/s, 95% CI: 0.03 to 0.25, *p* = 0.029; I^2^ = 67.45%; Figure 5A). Leave-one-out and intervention-duration subgroup analyses were considered exploratory and are presented in Supplementary Figures S13–S15.

**Figure 5:**
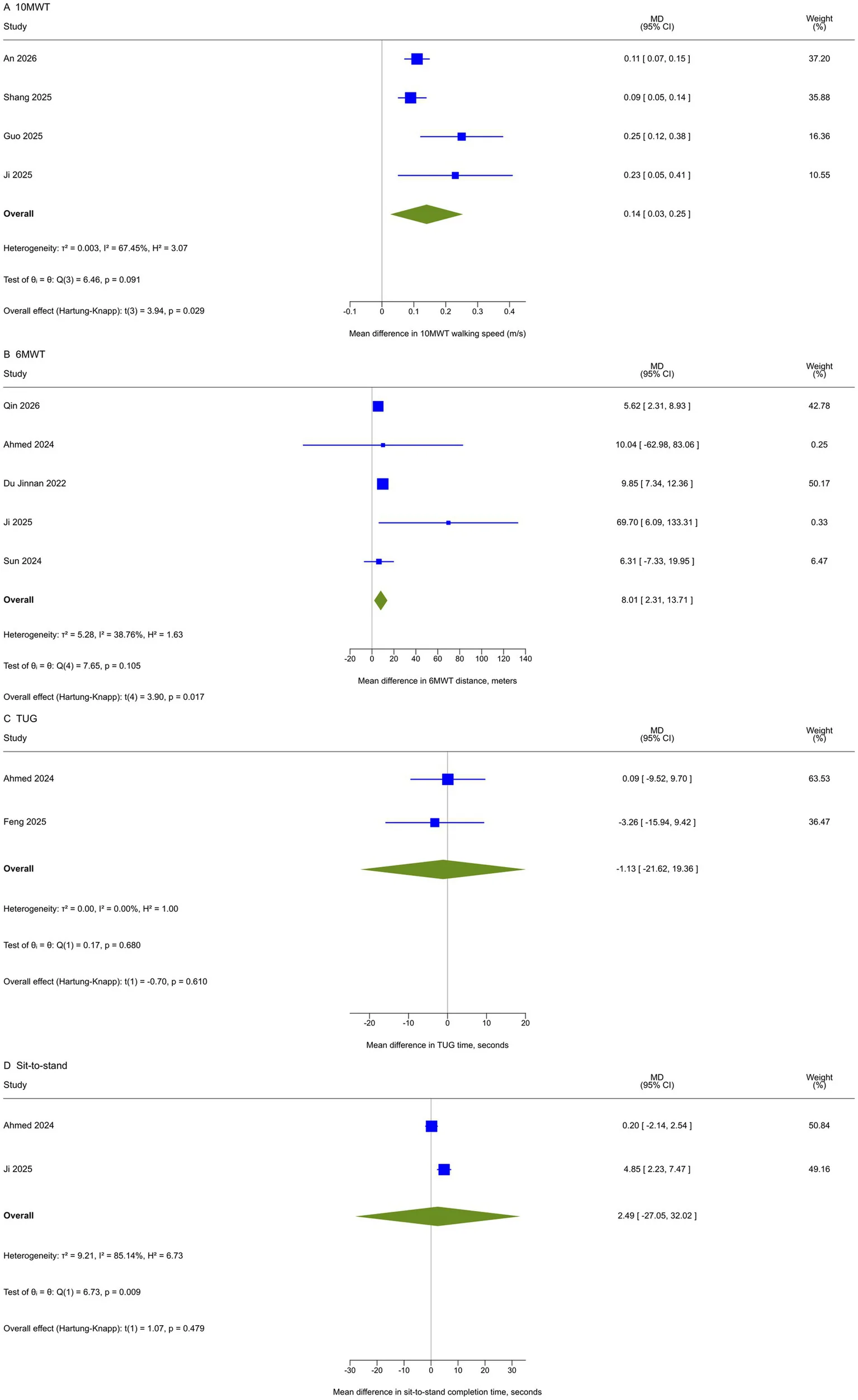
Forest plots of other functional outcomes: **(A)** knee extensor peak torque, **(B)** standard full-scale Barthel Index or Modified Barthel Index scores, **(C)** cadence, and **(D)** gait speed.

Three of the four studies explicitly used fast or maximal walking instructions, whereas the walking-speed instruction was unclear in one study. No study was clearly identified as using self-selected walking speed. A separate subgroup analysis by walking-speed condition was therefore not performed because the only alternative category contained a single study; however, differences in test implementation may have contributed to the observed heterogeneity.

For 10MWT walking speed, incorporation of two eligible grey-literature trials attenuated the pooled estimate and increased between-study heterogeneity (6 studies, 410 participants; MD = 0.09 m/s, 95% CI: −0.03 to 0.21, *p* = 0.102; I^2^ = 94.42%). Sensitivity A and Sensitivity B yielded identical results for this outcome because neither additional 10MWT result was excluded under the outcome-specific high-risk-of-bias rule. Corresponding grey-literature and timed-segment robustness analyses are shown in Supplementary Table S11 and Supplementary Figures S16, S17.

Five studies involving 271 participants assessed walking endurance using the 6MWT. BFRT increased walking distance by 8.01 m compared with control interventions (95% CI: 2.31 to 13.71, *p* = 0.017; I^2^ = 38.76%, τ^2^ = 5.28, H^2^ = 1.63; Figure 5B). Leave-one-out analyses showed pooled improvements ranging from 5.83 to 9.82 m, with all five estimates remaining statistically significant. The leave-one-out analysis is shown in Supplementary Figure S18.

For more complex functional mobility tasks, pooled effects were not statistically significant. Two studies involving 68 participants reported TUG outcomes, with no significant between-group difference (direction-aligned MD = −1.13 s, 95% CI: −21.62 to 19.36, *p* = 0.610; I^2^ = 0%; Figure 5C). Two studies involving 76 participants contributed 5TSTS/FTSTS completion-time data. The pooled direction-aligned effect was 2.49 s in favor of BFRT, but the confidence interval crossed the null (95% CI: −27.05 to 32.02, *p* = 0.479; I^2^ = 85.14%; Figure 5D). The broader three-study sit-to-stand analysis showed a similar favorable direction (SMD = 0.55, 95% CI: −0.02 to 1.12, *p* = 0.060), while the 30-s sit-to-stand test reported separately by Li et al. (11) showed a between-group difference of 2.80 repetitions (95% CI: −1.14 to 6.74). Overall, no clear effects were observed for TUG or sit-to-stand performance. The sit-to-stand leave-one-out analysis is shown in Supplementary Figure S19.

### Other functional outcomes

Other functional outcomes included knee extensor strength, activities of daily living, cadence, and gait speed.

Two studies involving 98 participants assessed knee extensor peak torque. A statistically significant pooled estimate favored BFRT (MD = 11.44 Nm, 95% CI: 6.56 to 16.32, *p* = 0.021), with no detected between-study heterogeneity (I^2^ = 0%, τ^2^ = 0.00; Figure 6A). Both contributing studies showed effects in the same direction; however, because only two studies contributed, this finding should be considered preliminary.

**Figure 6:**
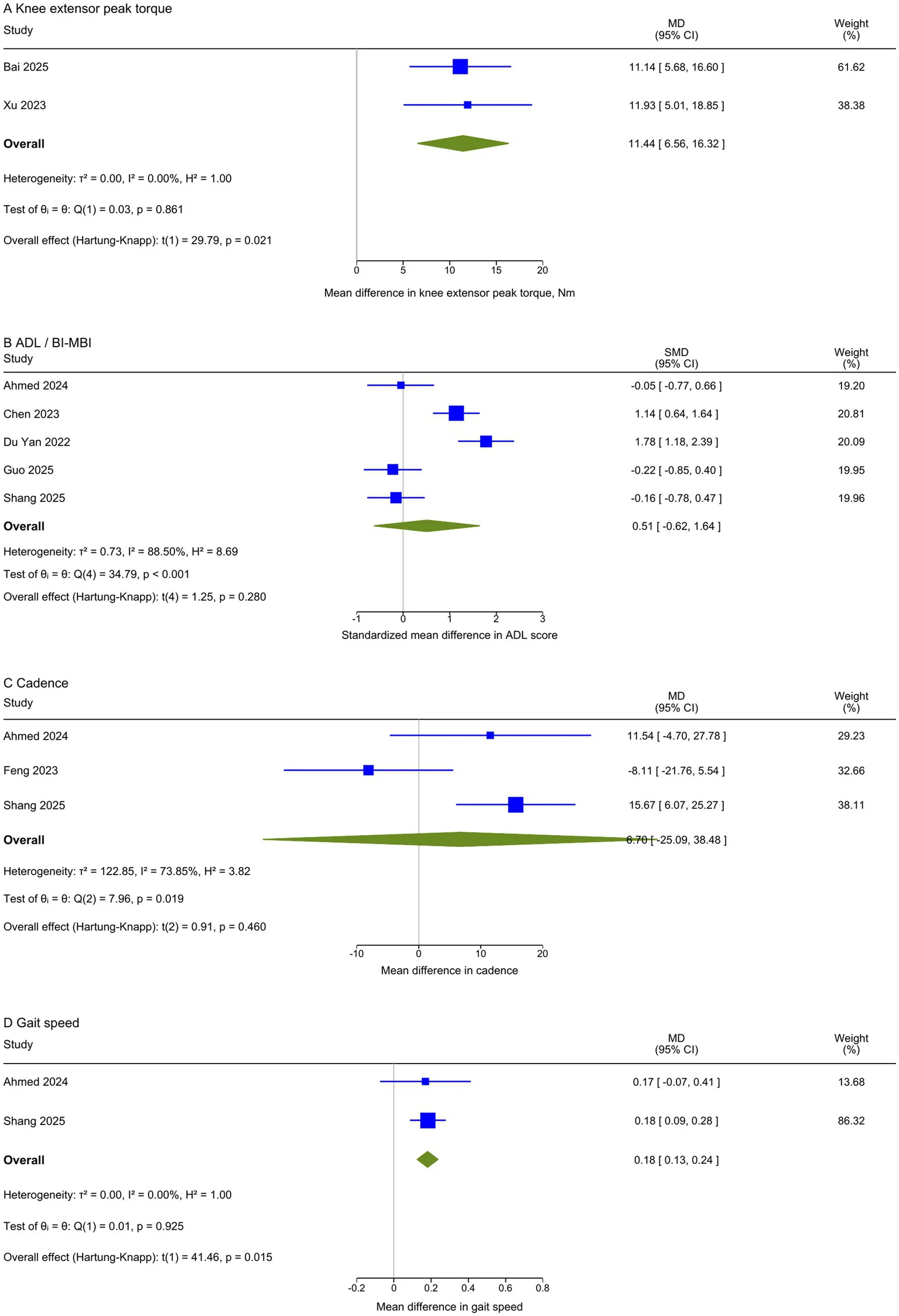
Forest plots of walking and functional mobility outcomes: **(A)** 10-meter walk test walking speed, **(B)** 6-min walk test distance, **(C)** Timed Up and Go completion time, and **(D)** five-times sit-to-stand completion time. For time-based outcomes, effect directions were aligned so that positive values indicated improvement in favor of blood flow restriction training.

Five studies involving 241 participants contributed standard full-scale BI or MBI data to the primary analysis of activities of daily living. The pooled effect favored BFRT numerically but was not statistically significant (SMD = 0.51, 95% CI: −0.62 to 1.64, *p* = 0.280; I^2^ = 88.5%, τ^2^ = 0.73; Figure 6B). When the analysis was broadened to include two additional studies reporting non-standard lower-limb ADL subscores, the pooled effect became statistically significant (7 studies; SMD = 0.66, 95% CI: 0.07 to 1.24, *p* = 0.028; I^2^ = 86.5%). The leave-one-out analysis is shown in Supplementary Figure S20.

An exploratory subgroup difference according to intervention duration was observed (*p* = 0.016), but this finding was not multiplicity-adjusted and should be interpreted cautiously. Studies lasting more than 4 weeks showed a pooled improvement of SMD = 0.99 (95% CI: 0.42 to 1.56; I^2^ = 81.0%), whereas studies lasting 4 weeks or less showed no significant improvement (SMD = −0.19, 95% CI: −0.63 to 0.25; I^2^ = 0%). No significant subgroup difference was observed according to BFRT training modality (*p* = 0.106). Corresponding subgroup analyses are shown in Supplementary Figures S21, S22.

Gait parameters showed different response patterns. Three studies involving 99 participants reported cadence, with no significant pooled between-group difference (MD = 6.70 steps/min, 95% CI: −25.09 to 38.48, *p* = 0.460; I^2^ = 73.85%; Figure 6C). The two contributing studies used different gait-speed assessment protocols. Ahmed et al. derived gait speed from a fastest-speed 10-m walk using video-based offline calculation, whereas Shang et al. used a GaitWatch instrumented 12-m habitual-speed walking assessment. Gait-speed values were harmonized to a common unit of m/s for pooling, but the underlying testing protocols were not standardized across studies. In contrast, a statistically significant pooled estimate from two studies involving 70 participants favored BFRT for gait speed (MD = 0.18 m/s, 95% CI: 0.13 to 0.24, *p* = 0.015; I^2^ = 0%; Figure 6D). Overall, preliminary two-study evidence favored BFRT for knee extensor strength and gait speed, whereas no clear pooled effects were observed for cadence or standard full-scale ADL. The cadence leave-one-out analysis is shown in Supplementary Figure S23.

### Safety

Adverse-event reporting was limited and inconsistent across the included trials. Among studies providing explicit safety information, no BFRT-related serious adverse event was reported. Reported BFRT-related events were generally mild and transient, including cuff-related discomfort, muscular pain, and localized ecchymosis or bruising; one participant receiving BFRT withdrew because of hypertension. No BFRT-related thrombotic event was reported. Detailed safety information is presented in Supplementary Table S12.

### Certainty of evidence

The certainty of evidence ranged from moderate to very low across outcomes. Evidence for the 6MWT was rated as moderate certainty. Evidence for FMA-LE, TUG, knee extensor peak torque, and gait speed was rated as low certainty, whereas evidence for BBS, 10MWT walking speed, ADL, 5TSTS/FTSTS, and cadence was rated as very low certainty. Downgrading was primarily due to risk of bias, inconsistency, and/or imprecision. For 10MWT walking speed, confidence was further reduced by the attenuation of the effect after inclusion of eligible grey literature. Full GRADE judgments are provided in Supplementary Table S8.

## Discussion

This systematic review and meta-analysis included 19 randomized controlled trials evaluating BFRT for lower-limb rehabilitation after stroke. Statistically significant pooled effects favored BFRT for FMA-LE, BBS, 6MWT, 10MWT walking speed, knee extensor peak torque, and gait speed, whereas no clear between-group effects were observed for TUG, sit-to-stand performance, cadence, or standard BI/MBI scores. However, the clinical importance of the significant findings was variable: the pooled FMA-LE and 6MWT effects were below commonly reported clinically important thresholds, the BBS estimate was close to an approximately 5-point reference, and the 10MWT estimate spanned effects below and above a reported 0.16 m/s threshold. The certainty of evidence varied across outcomes, with moderate-certainty evidence for the 6MWT and low- to very-low-certainty evidence for the remaining outcomes, reflecting differences in risk of bias, inconsistency, and imprecision. These findings indicate that statistically significant effects should be interpreted alongside their clinical magnitude and the certainty of the supporting evidence.

The magnitude of these pooled estimates provides additional clinical context. The 2.14-point FMA-LE difference was below reported MIC/MCID estimates of approximately 4–6 points, which vary across acute and chronic stroke populations and according to the perspective used to define meaningful change (24, 25). The 4.72-point BBS difference was close to the approximately 5-point MCID reported in some patients with early subacute stroke, although such thresholds may vary with recovery stage, baseline balance, and walking ability (26). For 10MWT walking speed, the pooled primary difference of 0.14 m/s was close to the 0.16 m/s MCID reported for comfortable gait speed in patients with subacute stroke and severe gait impairment. However, the 95% CI (0.03 to 0.25 m/s) included effects both below and above this reference value, and the threshold was derived from a specific stroke population and walking condition. The clinical importance of the pooled effect therefore remains uncertain. The 8.01-m increase in 6MWT distance was smaller than the approximately 34–71-m range reported across anchor definitions and baseline walking subgroups after stroke, while remaining directionally consistent with improved walking endurance (27). Taken together, although several pooled effects were statistically significant, their clinical relevance remains uncertain because some effect estimates were below or spanned clinically important thresholds, substantial heterogeneity was present for several outcomes, and the certainty of evidence was reduced by risk of bias and/or imprecision.

The absence of clear pooled effects for TUG and sit-to-stand performance should be interpreted cautiously. Each primary synthesis included only two studies, limiting the precision of the pooled estimates. For TUG, neither contributing trial demonstrated a significant between-group advantage of BFRT, and the pooled confidence interval was wide. For sit-to-stand performance, however, the individual study findings differed substantially. Ahmed et al. found little between-group difference in 5TSTST, whereas Ji et al. reported a significantly shorter FTSTS time with BFR-AT than with aerobic training alone (12.17 vs. 17.02 s), corresponding to an approximately 4.85-s between-group difference. This difference also exceeded the 2.81-s MCID reported for the five-times sit-to-stand test in stroke (28), although the applicability of this threshold across different stroke populations should be interpreted cautiously. The discordant study-level findings are consistent with the substantial heterogeneity in the pooled sit-to-stand analysis (I^2^ = 85.1%) and suggest that the inconclusive pooled estimate may reflect the small evidence base, imprecision, and between-study differences. The multi-component nature of TUG and sit-to-stand tasks may additionally limit the immediate transfer of improvements in lower-limb capacity to these functional outcomes, but it should not be considered the sole explanation for the non-significant pooled effects.

Compared with previous reviews, the present findings show how the evidence for BFRT in neurological rehabilitation has developed from an early and uncertain signal toward a more coherent pattern of lower-limb recovery after stroke. Vinolo-Gil et al. included only seven studies across heterogeneous neurological disorders and found no clear improvement in balance or lower-limb strength (13). Jønsson et al. subsequently identified 16 studies, but only four contributed efficacy data, leaving the functional effects of BFRT largely unresolved (14). More recently, Wang et al. synthesized 37 studies involving 963 participants across several neurological conditions and reported improvements in muscle strength, BBS, FMA-LE, and MBI, suggesting that the earlier absence of a balance effect was at least partly related to the limited evidence then available (15). Zhang et al. subsequently focused specifically on stroke and included 11 randomized trials with 522 participants. Their findings showed a pattern similar to ours: BBS and 6MWT improved, whereas TUG did not show a clear between-group effect (16). In the present analysis, BFRT increased BBS scores by 4.72 points and 6MWT distance by 8.01 m, while the pooled TUG effect remained non-significant. Across these successive reviews, a clinically recognizable pattern is emerging. BFRT appears to influence most directly the physical capacities that support locomotion, including paretic lower-limb motor control, force generation, postural stability, and walking endurance, whereas its effects on more complex mobility tasks are less consistent. The ADL findings add another important distinction. Wang et al. reported an overall improvement in MBI, whereas our stricter analysis limited to standard full-scale BI/MBI did not show a clear effect (15). When non-standard lower-limb ADL subscores were added, however, the pooled effect became significant. This suggests that some of the apparent ADL benefit in the existing literature may reflect improvement in lower-limb-dependent activities rather than a uniform gain in overall independence. Taken together, the evidence suggests a plausible sequence of functional response: BFRT may first improve lower-limb motor control, strength, balance, and basic walking capacity, while gains in higher-level function depend increasingly on whether these improvements are incorporated into repeated practice of sit-to-stand transitions, turning, transfers, stair negotiation, and everyday activities (29, 30).

Stroke stage varied substantially across the included trials and may have contributed to clinical heterogeneity. Participants ranged from early or subacute stroke to chronic ambulatory survivors, with corresponding differences in baseline function, recovery context, exercise tolerance, and comparator intensity (31, 32). However, stroke stage was not examined in the present subgroup analyses, and the available data do not support reliable stage-specific estimates of BFRT efficacy. Stroke stage should therefore be considered a plausible source of heterogeneity rather than a demonstrated effect modifier, and future adequately powered trials should directly evaluate whether responses to BFRT differ across stages of stroke recovery.

BFRT protocols varied substantially across trials, particularly in cuff application, pressure prescription, exercise modality and intensity, pressurization pattern, intervention duration, and comparator design. A key distinction was the use of fixed absolute pressures versus individualized prescriptions based on arterial occlusion pressure (AOP). The degree of vascular restriction produced by a given absolute pressure can vary according to cuff width, limb circumference, blood pressure, and individual vascular characteristics (33, 34). AOP-based prescription may provide a more standardized approach to BFRT dosing. Differences in exercise content, restriction pattern, treatment duration, and concomitant rehabilitation may also have contributed to between-study heterogeneity (35, 36). Accordingly, the pooled estimates should be interpreted as reflecting heterogeneous BFRT-assisted rehabilitation protocols rather than a single standardized intervention. Future trials should report cuff characteristics, pressure-prescription methods, exercise intensity, pressurization pattern, treatment frequency, and cumulative exposure in sufficient detail to improve reproducibility. Intervention duration may also contribute to heterogeneity, although the larger FMA-LE effect observed in studies lasting more than 4 weeks should be regarded as exploratory.

The secondary outcomes and physiological measures reported in the included trials provide a complementary explanation for the observed functional pattern. Preliminary evidence from two trials involving 98 participants favored BFRT for knee extensor peak torque by 11.44 Nm, with effects in the same direction and no detected between-study heterogeneity. This is clinically relevant because the quadriceps contribute directly to weight acceptance, knee stability during stance, transfers, and forward progression during walking (37). The walking outcomes were consistent with this finding. The primary 10MWT analysis showed a higher walking speed with BFRT, but this finding was not robust to the inclusion of eligible grey literature; 6MWT distance improved, and preliminary two-study evidence favored BFRT for gait speed, whereas cadence did not show a clear pooled improvement. Patients may therefore have improved their ability to generate and sustain force during walking without simply increasing step frequency. This pattern is also consistent with the physiological basis of BFRT. Proximal cuff inflation partially restricts arterial inflow and more substantially restricts venous return, accelerating local metabolite accumulation during otherwise low-load exercise (35, 38). As fatigue develops, additional motor-unit recruitment is required to maintain force output, while repeated exposure provides a metabolic and mechanical stimulus for neuromuscular and muscular adaptation (38, 39). Several included trials provide direct physiological clues. Bai et al. combined 30% 1RM resistance cycling with BFR and assessed FMA-LE, rectus femoris cross-sectional area, knee extensor peak torque, and cortical oxygenation using fNIRS, providing concurrent information on peripheral muscle performance and cortical activation (10). Li et al. assessed rectus femoris thickness and cross-sectional area together with gastrocnemius pennation angle after AOP-based BFR cycling, supporting the possibility of structural muscle adaptation during low-load training (11). Xu et al. reported changes in surface EMG activity of the affected rectus femoris, biceps femoris, tibialis anterior, and gastrocnemius together with changes in isokinetic muscle performance, linking BFRT with altered neuromuscular activation and force-generating capacity of the paretic limb (40). Chen et al. further examined appendicular muscle mass, skeletal muscle indices, growth hormone, IGF-1, and IL-6 during a longer BFRT program in older stroke patients with sarcopenia (41). Taken together, these studies suggest that BFRT may act across several related levels, increasing the metabolic demand of low-load exercise, enhancing recruitment of the paretic musculature, improving force production, and, with repeated training, promoting muscular adaptation (39). This framework is consistent with the pattern observed in the meta-analysis: effects were clearest for FMA-LE and walking endurance, with preliminary favorable estimates for knee extensor strength and gait speed, whereas higher-level tasks requiring rapid postural transitions, trunk control, bilateral coordination, turning, and task-specific motor strategies showed less consistent improvement.

We acknowledge several limitations of this review. First, the evidence base was uneven across outcomes. FMA-LE and BBS were supported by a larger number of trials, whereas TUG, sit-to-stand performance, knee extensor strength, gait speed, and cadence were informed by relatively few studies. Estimates for these outcomes are therefore less precise, and formal assessment of small-study effects was feasible only for FMA-LE. Most included trials were relatively small, and 18 of the 19 studies were conducted in China. In addition, differences in stroke stage, baseline functional status, rehabilitation setting, and treatment exposure may influence responses to BFRT. These characteristics may limit the generalizability of the pooled estimates across broader stroke populations, rehabilitation contexts, and healthcare settings. PROSPERO registration occurred after initiation of the review. In addition, the primary eligibility criteria were restricted to full-text journal reports and excluded grey literature, which may have limited the coverage of unpublished or non-journal evidence. Eligible grey-literature trials were therefore examined in additional sensitivity analyses for relevant outcomes. Because multiple outcomes and exploratory analyses were evaluated without formal multiplicity adjustment, an increased risk of type I error cannot be excluded, particularly for secondary and subgroup findings. Second, outcome definitions were not fully consistent across trials. This was particularly relevant for sit-to-stand performance and ADL, where completion-time measures, repetition-based tests, standard BI/MBI scores, and lower-limb-specific ADL subscores represented different functional constructs. Because most contributing 10MWT studies reported aggregate completion-time data rather than individual walking speeds, conversion to m/s required a group-level nonlinear approximation. Although study-specific timed distances were used, some approximation error in the transformed means and standard deviations cannot be excluded. Future BFRT trials would benefit from a more consistent outcome framework that distinguishes lower-limb motor impairment, muscle strength, balance, walking capacity, functional mobility, and global ADL. Objective measures such as dynamometry, instrumented gait analysis, and wearable activity monitoring could further complement clinical scales. Third, methodological limitations remained evident when risk of bias was considered at the outcome level. Among the 32 results contributing to the principal syntheses, most were judged to have some concerns, particularly because allocation concealment was frequently insufficiently reported and assessor blinding was unclear for several clinician-rated outcomes. These issues may be particularly relevant to outcomes such as FMA-LE, BBS, and BI/MBI, which involve a greater degree of assessor judgment than standardized timed or distance-based walking tests. Future trials should use adequately concealed allocation, blinded outcome assessment, prospective trial registration, and complete prespecified outcome reporting. Fourth, most trials focused on end-of-treatment effects, and longer-term follow-up was uncommon. Follow-up at 1, 3, and 6 months would help determine whether gains in motor function, balance, strength, and walking capacity are retained and whether they translate into community mobility, transfer independence, stair use, and daily physical activity. Finally, safety evaluation should become more systematic (42). No BFRT-related serious adverse event or thrombotic event was reported in studies providing explicit safety information, while reported events were generally mild and transient, including cuff discomfort, muscular pain, bruising, and one withdrawal because of hypertension. Future trials should prospectively record blood pressure responses, distal circulation, sensory symptoms, skin changes, suspected thrombotic events, premature cuff release, and treatment discontinuation, together with the frequency, duration, management, and relation of each event to BFRT (35, 42, 43). These improvements would allow future research to move beyond short-term efficacy and determine how reliably BFRT-induced gains in lower-limb capacity can be retained and translated into sustained functional recovery after stroke.

Complementary mechanistic and preclinical studies, including appropriate animal models where relevant, may further clarify the physiological and neurovascular mechanisms underlying BFRT after stroke and help inform the refinement of treatment parameters for subsequent clinical evaluation.

## Conclusion

This systematic review and meta-analysis suggests that BFRT may improve several lower-limb outcomes after stroke, particularly lower-limb motor function, balance, and walking endurance. The primary analysis also favored BFRT for 10MWT walking speed, although this effect was attenuated after eligible grey literature was incorporated. Favorable effects were also observed for knee extensor strength and gait speed, but these estimates were based on a limited number of studies, whereas no clear pooled effects were found for TUG, sit-to-stand performance, cadence, or standard BI/MBI. Overall, the findings support the potential role of BFRT as an adjunct to post-stroke rehabilitation, although the clinical importance of some effects remains uncertain and the certainty of evidence ranges from moderate to very low across outcomes. Further adequately powered, multicenter, well-designed trials should standardize key BFRT parameters, including cuff and pressure prescription, exercise dose, and treatment duration, and incorporate longer-term follow-up and systematic adverse-event monitoring to better define its clinical benefits, identify patient subgroups most likely to benefit, and improve the generalizability and consistency of BFRT application in stroke rehabilitation.

## Data Availability

The original contributions presented in the study are included in the article/Supplementary material, further inquiries can be directed to the corresponding author.
