# Supplementary material for: Blood flow restriction training for lower-limb function, walking and functional recovery after stroke: a systematic review and meta-analysis
Source: Front Neurol. 2026 Oct 26;17:1987031. doi: 10.3389/fneur.2026.1987031 (PMC13745224; doi:10.3389/fneur.2026.1987031)
Supplement: Supplementary file 1 [file Table_1.docx]

**Supplementary Material**

**Blood flow restriction training for lower-limb function, walking and functional recovery after stroke: a systematic review and meta-analysis**

**S1. Search strategy**

Search cutoff date: June 10, 2026.

**Table S1. Databases searched and records identified.**

| **Database** | **Records identified, n** | **Notes** |
| --- | --- | --- |
| **PubMed** | 63 | High-sensitivity Stroke AND BFRT search; full strategy below. |
| **Embase** | 114 | High-sensitivity Stroke AND BFRT search; full strategy below. |
| **Web of Science Core Collection** | 80 | Topic-field search; full strategy below. |
| **Cochrane Central Register of Controlled Trials (CENTRAL)** | 83 | Cochrane Library CENTRAL search; full strategy below. |
| **CNKI** | 90 | Initial retrieval before publication-type eligibility filtering. |
| **Wanfang** | 71 | Subject-field search; full strategy below. |
| **VIP** | 74 | Title/keyword-field search; full strategy below. |
| **SinoMed/CBM** | 14 | Common-fields search (title, abstract, keywords, and subject headings). |
| **Total** | 589 | Records identified from the eight databases before removal of records for other reasons and deduplication. |

Note. The initial CNKI search retrieved 90 records. Of these, 63 degree-thesis/grey-literature records were removed before title and abstract screening according to the predefined publication-type eligibility criteria, leaving 27 CNKI records in the screening dataset. Across all databases, 589 records were initially identified; after removal of these 63 records and 254 duplicates, 272 records proceeded to title and abstract screening.

**S1.1. PubMed**

(

"Stroke"[Mesh] OR "Cerebrovascular Disorders"[Mesh] OR "Brain Ischemia"[Mesh] OR

"Cerebral Infarction"[Mesh] OR "Intracranial Hemorrhages"[Mesh] OR "Hemiplegia"[Mesh] OR

stroke*[Title/Abstract] OR poststroke[Title/Abstract] OR post-stroke[Title/Abstract] OR

"cerebrovascular accident"[Title/Abstract] OR "cerebrovascular accidents"[Title/Abstract] OR

CVA[Title/Abstract] OR CVAs[Title/Abstract] OR "cerebrovascular apoplexy"[Title/Abstract] OR

"brain vascular accident"[Title/Abstract] OR "brain vascular accidents"[Title/Abstract] OR

"cerebrovascular stroke"[Title/Abstract] OR "cerebral stroke"[Title/Abstract] OR

"acute stroke"[Title/Abstract] OR "ischemic stroke"[Title/Abstract] OR

"ischaemic stroke"[Title/Abstract] OR "hemorrhagic stroke"[Title/Abstract] OR

"haemorrhagic stroke"[Title/Abstract] OR "intracerebral hemorrhage"[Title/Abstract] OR

"intracerebral haemorrhage"[Title/Abstract] OR "cerebral hemorrhage"[Title/Abstract] OR

"cerebral haemorrhage"[Title/Abstract] OR "brain infarction"[Title/Abstract] OR

"cerebral infarction"[Title/Abstract] OR hemiplegia[Title/Abstract] OR

hemiparesis[Title/Abstract] OR hemiparetic[Title/Abstract]

)

AND

(

"blood flow restriction"[Title/Abstract] OR "blood-flow restriction"[Title/Abstract] OR

"blood flow restricted"[Title/Abstract] OR "blood-flow-restricted"[Title/Abstract] OR

"blood flow restriction training"[Title/Abstract] OR "blood-flow restriction training"[Title/Abstract] OR

"blood flow restriction exercise"[Title/Abstract] OR "blood-flow restriction exercise"[Title/Abstract] OR

"blood flow restriction therapy"[Title/Abstract] OR "blood flow restriction resistance training"[Title/Abstract] OR

"low-load resistance training with blood flow restriction"[Title/Abstract] OR

"low intensity resistance training with blood flow restriction"[Title/Abstract] OR

"low-intensity resistance training with blood flow restriction"[Title/Abstract] OR

"vascular occlusion training"[Title/Abstract] OR "occlusion training"[Title/Abstract] OR

"blood flow occlusion"[Title/Abstract] OR KAATSU[Title/Abstract] OR "KAATSU training"[Title/Abstract] OR

BFR[Title/Abstract] OR BFRT[Title/Abstract] OR BFRE[Title/Abstract]

)

**S1.2. Embase**

(

'stroke'/exp OR 'cerebrovascular disease'/exp OR 'brain ischemia'/exp OR

'cerebral infarction'/exp OR 'intracranial hemorrhage'/exp OR 'hemiplegia'/exp OR

stroke*:ti,ab,kw OR poststroke:ti,ab,kw OR 'post-stroke':ti,ab,kw OR

'cerebrovascular accident*':ti,ab,kw OR cva:ti,ab,kw OR cvas:ti,ab,kw OR

'cerebrovascular apoplexy':ti,ab,kw OR 'brain vascular accident*':ti,ab,kw OR

'cerebrovascular stroke*':ti,ab,kw OR 'cerebral stroke*':ti,ab,kw OR

'acute stroke*':ti,ab,kw OR 'ischemic stroke*':ti,ab,kw OR 'ischaemic stroke*':ti,ab,kw OR

'hemorrhagic stroke*':ti,ab,kw OR 'haemorrhagic stroke*':ti,ab,kw OR

'intracerebral hemorrhage':ti,ab,kw OR 'intracerebral haemorrhage':ti,ab,kw OR

'cerebral hemorrhage':ti,ab,kw OR 'cerebral haemorrhage':ti,ab,kw OR

'brain infarction':ti,ab,kw OR 'cerebral infarction':ti,ab,kw OR

hemiplegia:ti,ab,kw OR hemiparesis:ti,ab,kw OR hemiparetic:ti,ab,kw

)

AND

(

'blood flow restriction':ti,ab,kw OR 'blood-flow restriction':ti,ab,kw OR

'blood flow restricted':ti,ab,kw OR 'blood-flow-restricted':ti,ab,kw OR

'blood flow restriction training':ti,ab,kw OR 'blood-flow restriction training':ti,ab,kw OR

'blood flow restriction exercise':ti,ab,kw OR 'blood-flow restriction exercise':ti,ab,kw OR

'blood flow restriction therapy':ti,ab,kw OR 'blood flow restriction resistance training':ti,ab,kw OR

'low-load resistance training with blood flow restriction':ti,ab,kw OR

'low intensity resistance training with blood flow restriction':ti,ab,kw OR

'low-intensity resistance training with blood flow restriction':ti,ab,kw OR

'vascular occlusion training':ti,ab,kw OR 'occlusion training':ti,ab,kw OR

'blood flow occlusion':ti,ab,kw OR kaatsu:ti,ab,kw OR 'kaatsu training':ti,ab,kw OR

bfr:ti,ab,kw OR bfrt:ti,ab,kw OR bfre:ti,ab,kw

)

**S1.3. Web of Science Core Collection**

TS=(

stroke* OR poststroke OR "post-stroke" OR "cerebrovascular accident*" OR CVA OR CVAs OR

"cerebrovascular apoplexy" OR "brain vascular accident*" OR "cerebrovascular stroke*" OR

"cerebral stroke*" OR "acute stroke*" OR "ischemic stroke*" OR "ischaemic stroke*" OR

"hemorrhagic stroke*" OR "haemorrhagic stroke*" OR "intracerebral hemorrhage" OR

"intracerebral haemorrhage" OR "cerebral hemorrhage" OR "cerebral haemorrhage" OR

"brain infarction" OR "cerebral infarction" OR "cerebrovascular disorder*" OR

hemiplegia OR hemiparesis OR hemiparetic

)

AND

TS=(

"blood flow restriction" OR "blood-flow restriction" OR "blood flow restricted" OR

"blood-flow-restricted" OR "blood flow restriction training" OR

"blood-flow restriction training" OR "blood flow restriction exercise" OR

"blood-flow restriction exercise" OR "blood flow restriction therapy" OR

"blood flow restriction resistance training" OR

"low-load resistance training with blood flow restriction" OR

"low intensity resistance training with blood flow restriction" OR

"low-intensity resistance training with blood flow restriction" OR

"vascular occlusion training" OR "occlusion training" OR "blood flow occlusion" OR

KAATSU OR "KAATSU training" OR BFR OR BFRT OR BFRE

)

**S1.4. Cochrane Library (CENTRAL)**

#1 MeSH descriptor: [Stroke] explode all trees

#2 MeSH descriptor: [Cerebrovascular Disorders] explode all trees

#3 MeSH descriptor: [Brain Ischemia] explode all trees

#4 MeSH descriptor: [Cerebral Infarction] explode all trees

#5 MeSH descriptor: [Intracranial Hemorrhages] explode all trees

#6 MeSH descriptor: [Hemiplegia] explode all trees

#7 (stroke* OR poststroke OR "post-stroke" OR "cerebrovascular accident*" OR CVA OR CVAs OR

"cerebrovascular apoplexy" OR "brain vascular accident*" OR "cerebrovascular stroke*" OR

"cerebral stroke*" OR "acute stroke*" OR "ischemic stroke*" OR "ischaemic stroke*" OR

"hemorrhagic stroke*" OR "haemorrhagic stroke*" OR "intracerebral hemorrhage" OR

"intracerebral haemorrhage" OR "cerebral hemorrhage" OR "cerebral haemorrhage" OR

"brain infarction" OR "cerebral infarction" OR hemiplegia OR hemiparesis OR hemiparetic):ti,ab,kw

#8 #1 OR #2 OR #3 OR #4 OR #5 OR #6 OR #7

#9 ("blood flow restriction" OR "blood-flow restriction" OR "blood flow restricted" OR

"blood-flow-restricted" OR "blood flow restriction training" OR

"blood-flow restriction training" OR "blood flow restriction exercise" OR

"blood-flow restriction exercise" OR "blood flow restriction therapy" OR

"blood flow restriction resistance training" OR

"low-load resistance training with blood flow restriction" OR

"low intensity resistance training with blood flow restriction" OR

"low-intensity resistance training with blood flow restriction" OR

"vascular occlusion training" OR "occlusion training" OR "blood flow occlusion" OR

KAATSU OR "KAATSU training" OR BFR OR BFRT OR BFRE):ti,ab,kw

#10 #8 AND #9

**S1.5. CNKI**

SU=('脑卒中' OR '卒中' OR '中风' OR '脑中风' OR '脑梗死' OR '脑梗塞' OR

'脑出血' OR '脑血管病' OR '脑血管疾病' OR '脑血管意外' OR '脑血管障碍' OR

'缺血性脑卒中' OR '出血性脑卒中' OR '脑梗死后' OR '脑卒中后' OR '偏瘫' OR '偏身瘫痪')

AND

SU=('血流限制' OR '血流限制训练' OR '血流限制性训练' OR '血流限制运动' OR

'血流限制性运动' OR '血流限制疗法' OR '血流限制治疗' OR '限制性血流训练' OR

'限制血流训练' OR '血流限制抗阻训练' OR '血流限制阻力训练' OR

'低负荷血流限制训练' OR '低强度血流限制训练' OR '加压训练' OR '加压运动' OR

'闭塞训练' OR '血管闭塞训练' OR 'KAATSU' OR 'BFR' OR 'BFRT' OR 'BFRE')

**S1.6. Wanfang**

(

主题:脑卒中 OR 主题:卒中 OR 主题:中风 OR 主题:脑中风 OR 主题:脑梗死 OR 主题:脑梗塞 OR

主题:脑出血 OR 主题:脑血管病 OR 主题:脑血管疾病 OR 主题:脑血管意外 OR

主题:缺血性脑卒中 OR 主题:出血性脑卒中 OR 主题:脑卒中后 OR 主题:偏瘫

)

AND

(

主题:血流限制 OR 主题:血流限制训练 OR 主题:血流限制性训练 OR

主题:血流限制运动 OR 主题:血流限制性运动 OR 主题:血流限制疗法 OR

主题:血流限制治疗 OR 主题:限制性血流训练 OR 主题:限制血流训练 OR

主题:血流限制抗阻训练 OR 主题:血流限制阻力训练 OR

主题:低负荷血流限制训练 OR 主题:低强度血流限制训练 OR

主题:加压训练 OR 主题:加压运动 OR 主题:闭塞训练 OR 主题:血管闭塞训练 OR

主题:KAATSU OR 主题:BFR OR 主题:BFRT OR 主题:BFRE

)

**S1.7. VIP**

(

题名或关键词=脑卒中 OR 题名或关键词=卒中 OR 题名或关键词=中风 OR

题名或关键词=脑中风 OR 题名或关键词=脑梗死 OR 题名或关键词=脑梗塞 OR

题名或关键词=脑出血 OR 题名或关键词=脑血管病 OR 题名或关键词=脑血管意外 OR

题名或关键词=缺血性脑卒中 OR 题名或关键词=出血性脑卒中 OR

题名或关键词=脑卒中后 OR 题名或关键词=偏瘫

)

AND

(

题名或关键词=血流限制 OR 题名或关键词=血流限制训练 OR

题名或关键词=血流限制性训练 OR 题名或关键词=血流限制运动 OR

题名或关键词=血流限制性运动 OR 题名或关键词=血流限制疗法 OR

题名或关键词=血流限制治疗 OR 题名或关键词=限制性血流训练 OR

题名或关键词=限制血流训练 OR 题名或关键词=血流限制抗阻训练 OR

题名或关键词=血流限制阻力训练 OR 题名或关键词=低负荷血流限制训练 OR

题名或关键词=低强度血流限制训练 OR 题名或关键词=加压训练 OR

题名或关键词=加压运动 OR 题名或关键词=闭塞训练 OR

题名或关键词=血管闭塞训练 OR 题名或关键词=KAATSU OR

题名或关键词=BFR OR 题名或关键词=BFRT OR 题名或关键词=BFRE

)

**S1.8. SinoMed/CBM**

Search fields: common fields (Chinese title, abstract, keywords, and subject headings).

#1 脑卒中 OR 卒中 OR 中风 OR 脑中风 OR 脑梗死 OR 脑梗塞 OR 脑出血 OR

脑血管病 OR 脑血管疾病 OR 脑血管意外 OR 脑血管障碍 OR

缺血性脑卒中 OR 出血性脑卒中 OR 脑卒中后 OR 偏瘫 OR 偏身瘫痪

#2 血流限制 OR 血流限制训练 OR 血流限制性训练 OR 血流限制运动 OR

血流限制性运动 OR 血流限制疗法 OR 血流限制治疗 OR 限制性血流训练 OR

限制血流训练 OR 血流限制抗阻训练 OR 血流限制阻力训练 OR

低负荷血流限制训练 OR 低强度血流限制训练 OR 加压训练 OR 加压运动 OR

闭塞训练 OR 血管闭塞训练 OR KAATSU OR BFR OR BFRT OR BFRE

#3 #1 AND #2

**Search-strategy note**

The strategy intentionally did not include lower-extremity, muscle-strength, muscle-morphology, balance, gait, FMA-LE, BBS, ADL, or randomized controlled trial terms. These restrictions were avoided because they could reduce sensitivity and exclude otherwise eligible BFRT studies. Eligibility criteria were applied during deduplication and screening rather than within the database query.

**S2. Data harmonization and statistical handling**

**Table S2. Data harmonization and analytical handling for quantitative synthesis.**

| **Issue** | **Analytical_handling** | **Rationale** |
| --- | --- | --- |
| Direction of effect | For time-to-complete outcomes, effect direction was aligned so that positive pooled values indicate improvement in favor of BFRT. | This provides a consistent clinical direction across outcomes. |
| FMA-LE construct | Only lower-extremity Fugl-Meyer outcomes representing the lower-extremity construct were eligible for FMA-LE pooling. | Total FMA scores (0–100) were not treated as FMA-LE. |
| Luo 2022 FMA-LE | The Luo 2022 FMA-LE endpoint was excluded from the revised primary FMA-LE model and re-included in sensitivity analysis. | The reported post-treatment mean exceeded the standard 34-point FMA-LE range and the study was highly influential in diagnostic analyses. |
| Li 2025 digitized FMA-LE | Figure-digitized FMA-LE data were retained in the revised primary analysis and a sensitivity analysis additionally excluding Li 2025 was performed. | This evaluates whether the pooled estimate depends on figure-derived data. |
| ADL harmonization | The stricter primary ADL analysis uses studies reporting standard full BI/MBI constructs. A broader SMD sensitivity analysis additionally includes non-standard lower-limb ADL/MBI subscores. | This avoids treating non-equivalent ADL scale ranges as the same clinical construct while preserving a broader sensitivity analysis. |
| Qin 2026 ADL subscore | The lower-limb MBI/ADL subscore was not included in the stricter standard BI/MBI primary set and was retained only in the broader SMD sensitivity analysis. | The reported subscore construct/range is non-standard and contains a source-level range inconsistency. |
| Du Jinnan 2022 ADL subscore | The lower-limb-related MBI subset was not included in the stricter standard BI/MBI primary set and was retained only in the broader SMD sensitivity analysis. | The reported construct is not equivalent to a standard full BI/MBI total score. |
| Sit-to-stand outcomes | 5TSTS/FTSTS completion-time outcomes were pooled together. The 30-s sit-to-stand repetition outcome was reported separately; the previous three-study SMD synthesis is retained only as an exploratory broad sensitivity analysis. | Completion time and number of repetitions represent related but non-identical tasks with different units and directions. |
| 10MWT walking-speed conversion | For studies reporting completion time, walking speed was approximated as actual timed distance divided by group mean completion time; SDspeed was approximated by the first-order delta method. Study-specific timed distances were used, and eligible multi-arm BFRT groups were transformed separately before standard combination. | This expresses 10MWT results in m/s while preserving the timed segment reported by each study and avoiding double counting of shared controls. |
| 10MWT walking-speed condition | An 2026 and Shang 2025 used fast walking instructions, Ji 2025 used maximal walking, and the walking-speed instruction in Guo 2025 was unclear. | A separate subgroup analysis was not conducted because three studies used fast/maximal walking and the remaining study had an unclear instruction rather than a confirmed self-selected condition. |
| Gait speed | Ahmed 2024 assessed gait speed during a fastest-speed 10-m walk using video-based offline calculation, whereas Shang 2025 used a GaitWatch instrumented 12-m habitual-speed walking assessment. Gait-speed values were harmonized to m/s before pooling, but the underlying walking instructions and measurement protocols were not otherwise standardized. | This permits pooling using a common unit while acknowledging differences in gait-testing conditions. |
| An 2026 multi-arm trial | The 40%, 50%, and 60% AOP BFRT groups were combined into one BFRT group using standard formulas for combined sample size, mean, and standard deviation; the shared control group was used once. | This prevents unit-of-analysis error and double counting of the shared control group. |
| Post-intervention time point | The prespecified end-of-treatment time point was used for the primary pooled analysis unless an outcome was explicitly analyzed at another prespecified time point. | This maintains consistency across studies with repeated assessments. |
| Yang 2021 FMA | The reported total FMA score (0–100) was not included in FMA-LE pooling. | The total FMA construct is not equivalent to the standard lower-extremity FMA subscale. |

**S3. Outcome-specific risk of bias assessment**

**Table S3. Outcome-specific risk-of-bias judgments for the principal outcomes using the Cochrane RoB 2 tool.**

| **Outcome** | **Study** | **D1** | **D2** | **D3** | **D4** | **D5** | **Overall** |
| --- | --- | --- | --- | --- | --- | --- | --- |
| **FMA-LE** | Qin 2026 | Low risk | Low risk | Low risk | Low risk | Low risk | **Low risk** |
| **FMA-LE** | An 2026 | Some concerns | Low risk | Low risk | Low risk | Low risk | **Some concerns** |
| **FMA-LE** | Bai 2025 | Some concerns | Low risk | Some concerns | Low risk | Some concerns | **Some concerns** |
| **FMA-LE** | Li 2025 | Some concerns | Low risk | Low risk | Low risk | Low risk | **Some concerns** |
| **FMA-LE** | Feng 2025 | Low risk | Low risk | Low risk | Low risk | Low risk | **Low risk** |
| **FMA-LE** | Guo 2025 | Some concerns | Low risk | Low risk | Some concerns | Low risk | **Some concerns** |
| **FMA-LE** | Shang 2025 | Some concerns | Low risk | Low risk | Some concerns | Low risk | **Some concerns** |
| **FMA-LE** | Du Yan 2022 | Some concerns | Low risk | Low risk | Some concerns | Low risk | **Some concerns** |
| **FMA-LE** | Du Jinnan 2022 | Some concerns | Low risk | Low risk | Some concerns | Low risk | **Some concerns** |
| **FMA-LE** | Xu 2023 | Some concerns | Low risk | Low risk | Some concerns | Low risk | **Some concerns** |
| **BBS** | Qin 2026 | Low risk | Low risk | Low risk | Low risk | Low risk | **Low risk** |
| **BBS** | An 2026 | Some concerns | Low risk | Low risk | Low risk | Low risk | **Some concerns** |
| **BBS** | Gong 2025 | Some concerns | Low risk | Low risk | Some concerns | Low risk | **Some concerns** |
| **BBS** | Guo 2025 | Some concerns | Low risk | Low risk | Some concerns | Low risk | **Some concerns** |
| **BBS** | Sun 2024 | Some concerns | Low risk | Low risk | Low risk | Low risk | **Some concerns** |
| **BBS** | Tang 2025 | Some concerns | Low risk | Low risk | Some concerns | Low risk | **Some concerns** |
| **BBS** | Yang 2021 | Some concerns | Low risk | Low risk | Some concerns | Some concerns | **Some concerns** |
| **BBS** | Du Yan 2022 | Some concerns | Low risk | Low risk | Some concerns | Low risk | **Some concerns** |
| **10MWT** | An 2026 | Some concerns | Low risk | Low risk | Low risk | Low risk | **Some concerns** |
| **10MWT** | Guo 2025 | Some concerns | Low risk | Low risk | Low risk | Low risk | **Some concerns** |
| **10MWT** | Ji 2025 | Some concerns | Low risk | Low risk | Low risk | Low risk | **Some concerns** |
| **10MWT** | Shang 2025 | Some concerns | Low risk | Low risk | Low risk | Low risk | **Some concerns** |
| **6MWT** | Qin 2026 | Low risk | Low risk | Low risk | Low risk | Low risk | **Low risk** |
| **6MWT** | Ahmed 2024 | Low risk | Low risk | Low risk | Low risk | Low risk | **Low risk** |
| **6MWT** | Du Jinnan 2022 | Some concerns | Low risk | Low risk | Low risk | Low risk | **Some concerns** |
| **6MWT** | Ji 2025 | Some concerns | Low risk | Low risk | Low risk | Low risk | **Some concerns** |
| **6MWT** | Sun 2024 | Some concerns | Low risk | Low risk | Low risk | Low risk | **Some concerns** |
| **BI/MBI** | Ahmed 2024 | Low risk | Low risk | Low risk | Low risk | Low risk | **Low risk** |
| **BI/MBI** | Chen 2023 | Some concerns | Low risk | Low risk | Some concerns | Low risk | **Some concerns** |
| **BI/MBI** | Du Yan 2022 | Some concerns | Low risk | Low risk | Some concerns | Low risk | **Some concerns** |
| **BI/MBI** | Guo 2025 | Some concerns | Low risk | Low risk | Some concerns | Low risk | **Some concerns** |
| **BI/MBI** | Shang 2025 | Some concerns | Low risk | Low risk | Some concerns | Low risk | **Some concerns** |

Abbreviations: D1, bias arising from the randomization process; D2, bias due to deviations from intended interventions; D3, bias due to missing outcome data; D4, bias in measurement of the outcome; D5, bias in selection of the reported result; FMA-LE, Fugl-Meyer Assessment of the Lower Extremity; BBS, Berg Balance Scale; 10MWT, 10-meter walk test; 6MWT, 6-minute walk test; BI, Barthel Index; MBI, Modified Barthel Index.

Judgments were made for specific outcome results contributing to the principal meta-analyses. Accordingly, a single trial may appear more than once when it contributed data to more than one principal outcome.

**Table S4. Reports excluded after full-text assessment and reasons for exclusion.**

| **First author, year** | **Report title** | **Publication type** | **Reason for exclusion from the primary review** | **Post hoc handling** |
| --- | --- | --- | --- | --- |
| Deng, 2024 | Effects of blood-flow-restricted resistance cycling on lower-limb motor function and balance after stroke | Master’s thesis | Excluded under the prespecified publication-type criterion because the primary review was restricted to full-text journal articles. | Independent eligible randomized trial; included in the post hoc grey-literature sensitivity analysis. |
| Feng, 2023 | Effects of blood flow restriction combined with exercise training on physical function and gait in patients with stroke | Master’s thesis | Excluded under the prespecified publication-type criterion. | Linked/duplicate report of the corresponding published journal trial; not counted as an independent trial. |
| Lan, 2024 | Effects of blood flow restriction combined with electromyographic biofeedback on lower-limb function in patients with post-stroke hemiplegia | Master’s thesis | Excluded under the prespecified publication-type criterion because the primary review was restricted to full-text journal articles. | Independent eligible randomized trial; included in the post hoc grey-literature sensitivity analysis. |
| Liu, 2021 | Effects of blood flow restriction combined with isokinetic strength training on lower-limb function in patients with hemiplegia | Master’s thesis | Excluded under the prespecified publication-type criterion because the primary review was restricted to full-text journal articles. | Independent eligible randomized trial; included in the post hoc grey-literature sensitivity analysis. |
| Tang, 2022 | Effects of blood flow restriction training on balance function in patients with ischemic stroke | Master’s thesis | Excluded under the prespecified publication-type criterion. | Earlier/duplicate report of the subsequently published journal article; not counted as an independent trial. |
| Wen, 2024 | Effects of blood flow restriction on lower-limb motor function and muscle thickness in patients with hemiplegia | Master’s thesis | Excluded under the prespecified publication-type criterion. | Potentially overlapping report within the Feng trial family; not treated as an independent trial. |
| Wu and Tang, 2024 | Effects of blood flow restriction combined with isokinetic strength training on lower-limb function in patients with hemiplegia | Conference proceeding | Excluded under the prespecified publication-type criterion because conference proceedings were not eligible for the primary review. | Not included in quantitative sensitivity analysis because sufficiently extractable arm-level outcome data were unavailable. |
| Xu, 2022 | Effects of blood flow restriction combined with low-intensity resistance training on lower-limb strength, function, and surface electromyography after stroke | Master’s thesis | Excluded under the prespecified publication-type criterion. | Earlier/duplicate report of the subsequently published journal article; not counted as an independent trial. |
| Yue, 2023 | Effects of low-load blood flow restriction training on balance function in patients with posterior-circulation ischemic stroke | Master’s thesis | Excluded under the prespecified publication-type criterion because the primary review was restricted to full-text journal articles. | Independent eligible randomized trial; included in the post hoc grey-literature sensitivity analysis. |
| Zhang, 2018 | Effects of blood flow restriction combined with neuromuscular electrical stimulation on lower-limb function after stroke | Master’s thesis | Excluded under the prespecified publication-type criterion because the primary review was restricted to full-text journal articles. | Independent eligible randomized trial; included in the post hoc grey-literature sensitivity analysis. |

Note. Titles of Chinese-language theses and conference reports are translated into English for readability. Exclusion refers to the prespecified eligibility criteria for the primary review. Eligible independent grey-literature randomized trials were subsequently evaluated in post hoc sensitivity analyses and were not incorporated into the primary meta-analysis. Reports judged to represent duplicate or potentially overlapping trial cohorts were not counted independently.

**S4. Characteristics of included studies and BFRT protocols**

**Table S5A. Core study and participant characteristics.**

| **Study** | **Country** | **Design** | **Sample randomized / analyzed** | **Age** | **Sex** | **Stroke type** | **Stroke stage / time since stroke** | **Lesion / clinical phenotype** | **Baseline clinical status** |
| --- | --- | --- | --- | --- | --- | --- | --- | --- | --- |
| Ahmed 2024 | Turkey | Two-arm, single-blinded randomized controlled trial. | Randomized: 32 total; LIRT-BFR 16; HIRT 16; Analyzed: 30 total; 15/15. | LIRT-BFR 54.20±13.19 y; HIRT 54.73±14.08 y. | LIRT-BFR 10M/5F; HIRT 9M/6F among analyzed participants. | Ischemic stroke survivors. Baseline table additionally classifies stage as acute/subacute vs chronic: 7/8 LIRT-BFR and 6/9 HIRT. | Time since stroke 25.60±29.89 mo LIRT-BFR; 23.06±31.45 mo HIRT. | Ischemic stroke with lower-extremity motor impairment; paretic side right 8/15 vs 9/15, left 7/15 vs 6/15; LIRT-BFR right 8/15, left 7/15; HIRT right 9/15, left 6/15. | FAC 4.0±0.84 LIRT-BFR and 3.93±0.70 HIRT; walking aid used by 6/15 vs 8/15. |
| An 2026 | China | Four-arm randomized controlled trial. | Randomized: 200 total; 50/group; Analyzed: 196 total; 49/group after one dropout in each arm. | Control 66.88±5.11; 40%AOP 66.44±4.94; 50%AOP 66.86±5.48; 60%AOP 68.78±5.09 y. | M/F: control 25/25; 40%AOP 27/23; 50%AOP 25/25; 60%AOP 22/28. | Elderly ischemic stroke with frailty. | Disease duration (d): 15.10±1.16; 15.30±1.12; 15.60±1.12; 15.28±0.94. | Ischemic stroke. Lesion territory LACI/POCI/PACI: control 29/3/18; 40%AOP 24/6/20; 50%AOP 27/6/17; 60%AOP 24/5/21. | Elderly patients with frailty; FFP measured; baseline motor variables comparable. |
| Bai 2025 | China | Prospective, three-arm, double-blind randomized clinical trial with fNIRS. | Randomized: 66 enrolled; Analyzed: 60 completed/analyzed: BFR 20; low-load (LL) 20; high-load (HL) 20. | BFR 61.7±9.6 y; LL 60.9±10.3 y; HL 61.0±8.0 y. | Male: BFR 12/20; LL 11/20; HL 12/20. | Ischemic stroke only. | First subcortical ischemic stroke, 2 weeks–6 months after onset. Baseline table reports time poststroke BFR 23.0 (27.0), LL 29.0 (46.0), HL 27.5 (54), unit not printed in extracted table. | Unilateral supratentorial subcortical ischemic lesion; large cortical lesions and severe carotid/cerebral stenosis excluded; Affected hemiplegia left: BFR 15/20; LL 12/20; HL 13/20. | Lower-extremity major flexor/extensor MMT ≥3; MMSE BFR 23.8±4.2, LL 24.2±4.0, HL 25.7±4.0. |
| Chen 2023 | China | Two-arm randomized controlled clinical study. | Randomized: 71 total; BFR+LIRT 35; LIRT 36; Analyzed: 71; all completed the 12-week training. | BFR+LIRT 65.8±10.2 y; control 66.9±11.7 y. | BFR+LIRT 22M/13F; control 21M/15F. | First-ever unilateral cerebral infarction with sarcopenia. | Disease duration <3 months; BFR+LIRT 32.3±5.5 d; control 31.6±6.4 d. | Unilateral new cerebral infarction confirmed by CT/MRI; elderly patients with sarcopenia and hemiplegia. | Lower-limb Brunnstrom ≥III; modified Ashworth <II; AWGS 2019 sarcopenia criteria. |
| Du Jinnan 2022 | China | Two-arm randomized clinical study. | Randomized: 60 total; 30/30; Analyzed: 60 total; 30/30. | BFR+rTMS 52.1±3.9 y; control rTMS 51.8±4.2 y. | BFR+rTMS 17M/13F; control 18M/12F. | Stroke diagnosed by CT/MRI; subtype distribution NR. | NR | Stroke with lower-limb dysfunction; conscious, stable vital signs, no cognitive disorder; no prior stroke history. | Conscious, stable, no cognitive impairment. |
| Du Yan 2022 | China | Two-arm randomized controlled clinical study. | Randomized: 60 total; 30/30; Analyzed: 60 total; 30/30. | BFR+resistance 66.8±11.2 y; control 67.2±10.8 y. | BFR 18M/12F; control 17M/13F. | BFR ischemic/hemorrhagic 23/7; control 21/9. | BFR 76.4±18.4 d; control 75.6±11.1 d. | Elderly stroke with hemiplegia; BFR left/right 14/16; control 13/17. | Age≥60; lower-limb motor impairment; detailed criteria in article. |
| Feng 2023 | China | Two-arm randomized controlled clinical trial. | Randomized: 34 total; 17/17; Analyzed: 29 total; BFR-ET 15; ET 14. | BFR-ET 54.07±10.44 y; ET 45.29±13.74 y. | BFR-ET 10M/5F; ET 11M/3F. | BFR-ET ischemic 5/15, hemorrhagic 10/15; ET ischemic 4/14, hemorrhagic 10/14. | Time since stroke 12.27±11.55 mo BFR-ET; 14.36±15.45 mo ET. | Stroke with hemiplegic lower-limb dysfunction and impaired gait stability. | Lower-limb Brunnstrom II–V; some walking ability under supervision; MMSE 26.40±3.58 vs 23.43±5.96. |
| Feng 2025 | China | Randomized, assessor-blinded, block-randomized controlled clinical trial. | Randomized: 40 total; BFRET 20; ET 20; Analyzed: Flow diagram: post-treatment BFRET 20, ET 18 (38); gait analysis BFRET 19, ET 17 (36). Narrative text also states 36 post-treatment cases, creating an internal discrepancy. | BFRET 52.70±11.12 y; ET 44.85±12.99 y. | 15M/5F in each randomized group. | BFRET ischemic 7/20, hemorrhagic 13/20; ET ischemic 4/20, hemorrhagic 16/20. | Time from stroke to baseline median 7.0 mo (IQR 4.0–23.5) BFRET and 6.0 mo (3.5–24.0) ET. | Unilateral hemispheric stroke; ischemic or hemorrhagic. | Lower-limb Brunnstrom > grade II; MMSE median 28 (26–29) BFRET and 25 (19–29) ET. |
| Gong 2025 | China | Two-arm randomized clinical study. | Randomized: 52 total; 26/26; Analyzed: 52 total; 26/26. | Control 61.69±9.10 y; BFR 57.50±12.17 y. | Control 13M/13F; BFR 17M/9F. | Control infarction 21/hemorrhage 5; BFR infarction 22/hemorrhage 4. | Within 6 months; reported disease duration 26.19±4.60 d control and 27.42±6.73 d BFR. | Stroke with lower-limb weakness/balance impairment. | MMSE >25; lower-limb Brunnstrom ≥III; MMT≥2; Ashworth<2; standing balance≥1; able to walk with supervision or independently. |
| Guo 2025 | China | Two-arm randomized controlled clinical study. | Randomized: 40 total; suspension 20; BFR+suspension 20; Analyzed: 40 total. | Suspension 62.95±7.26 y; combined 62.15±7.36 y. | Suspension 14M/6F; combined 13M/7F. | Suspension ischemic/hemorrhagic 14/6; combined 16/4. | Disease duration 11.65±4.34 weeks suspension; 12.00±3.92 weeks combined. | Stroke with knee hyperextension; CT/MRI-confirmed cerebral hemorrhage or infarction; Suspension left/right 14/6; combined 11/9. | Age 35–77; MMSE≥24; Brunnstrom II–V; Holden≥1. |
| Ji 2025 | China | Two-arm randomized controlled clinical study. | Randomized: 46 total; aerobic 23; BFR-AT 23; Analyzed: 46 total; 23/23. | Aerobic 62.13±10.44 y; BFR-AT 62.43±12.05 y. | Aerobic 16M/7F; BFR-AT 15M/8F. | Aerobic hemorrhage/infarction 6/17; BFR-AT 9/14. | First unilateral stroke 1–3 months by inclusion criteria; duration 54.52±23.24 d vs 51.35±19.05 d. | Unilateral lesion; able to stand/walk; Aerobic left/right 13/10; BFR-AT 14/9. | Conscious; no major sensory/vision/hearing impairment; able to stand and walk independently; age 18–75. |
| Li 2025 | China | Two-arm randomized controlled study. | Randomized: 34 total; 17/17; Analyzed: 34 total; 17 control and 17 experimental. | Control 64.41±12.90 y; BFR 59.59±8.95 y. | Control 15M/2F; BFR 14M/3F. | Control: cerebral infarction 6, cerebral hemorrhage 11; BFR: infarction 8, hemorrhage 9. | Onset <6 months; disease duration control 41.53±33.50 d, BFR 47.06±42.26 d. | CT/MR-confirmed stroke; mixed ischemic/hemorrhagic; Control left 15/right 2; BFR left 14/right 3. | Seated balance level 2 required. |
| Luo 2022 | China | Two-arm randomized controlled clinical study. | Randomized: 40 total; 20/20; Analyzed: 40 total; 20/20. | Control 46.81±2.36 y (range 22–72); experimental 54.03±2.55 y (34–75). | Control 14M/6F; experimental 11M/9F. | Stroke with lower-limb dysfunction; subtype not reported in baseline characteristics. | NR | Post-stroke lower-limb dysfunction. | Able to understand and communicate, high adherence, able to tolerate pressurized training. |
| Qin 2026 | China | Parallel-group, two-arm, assessor-blinded randomized controlled trial; repeated-measures design. | Randomized: Source is internally inconsistent: methods/flow diagram indicate 82 randomized (41/41), whereas abstract states 80 enrolled; Analyzed: 80 total: RR 40; RR+BFRT 40. | RR 58.90±7.775 y; RR+BFRT 57.60±7.980 y. | RR 30M/10F; RR+BFRT 32M/8F. | Ischemic: 12/40 RR, 18/40 RR+BFRT; hemorrhagic: 28/40 RR, 22/40 RR+BFRT. | Disease duration 45.7±14.6 d RR and 45.9±13.9 d RR+BFRT. | CT/MRI-confirmed ischemic or hemorrhagic stroke with hemiplegia; cerebellar/vestibular impairments causing balance dysfunction excluded; Right 19/40 RR, 16/40 RR+BFRT; left 21/40 RR, 24/40 RR+BFRT. | MMSE 21.88±3.70 RR and 20.90±4.10 RR+BFRT; clinically stable vital signs required. |
| Shang 2025 | China | Two-arm randomized controlled clinical study. | Randomized: 40 total; 20 suspension; 20 BFR+suspension; Analyzed: 40; no dropout. | Suspension 54.27±7.18 y; combined 54.40±6.86 y. | Suspension 14M/6F; combined 16M/4F. | Suspension ischemic/hemorrhagic 14/6; combined 15/5. | Duration 8.20±1.82 weeks suspension; 8.67±1.72 weeks combined. | Stroke confirmed by CT/MRI; hemiplegic lower-limb dysfunction; Suspension left/right 11/9; combined 12/8. | Lower-limb Brunnstrom II–V; Holden walking ability ≥1; age 40–70. |
| Sun 2024 | China | Three-arm randomized controlled clinical study with follow-up. | Randomized: Report is internally ambiguous: 90 were enrolled with 10 described as dropouts, while methods then state 80 were randomized into three final groups; Analyzed: 80 total: combined WBV+BFR 27; WBV 28; control 25. | Combined 70.70±4.91; WBV 69.93±5.89; control 71.32±4.21 y. | Combined 15M/12F; WBV 18M/10F; control 18M/7F. | Combined infarction/hemorrhage 18/9; WBV 20/8; control 18/7. | Rehabilitation phase, <6 months; duration 42.63±21.48 d combined, 51.75±26.56 d WBV, 46.16±22.97 d control. | Hemiplegic stroke; cerebellar and brainstem infarction excluded; Combined left/right 17/10; WBV 19/9; control 16/9. | Age 60–85; standing balance 2–3; independent or cane-assisted walking ≥30 m; MMSE >21. |
| Tang 2025 | China | Two-arm randomized clinical study. | Randomized: 32 total; 16/16; Analyzed: 32 total; 16/16. | BFR 52±12 y; control 52±9 y. | BFR 13M/3F; control 14M/2F. | Unilateral basal-ganglia ischemic stroke only. | First onset within 8 weeks; BFR 32±12 d; control 27±14 d. | Unilateral basal-ganglia cerebral infarction confirmed by MRI; BFR left/right 9/7; control 9/7. | Sitting balance grade 2; standing balance ≥2; Brunnstrom≥3; modified Ashworth≤1+; age 30–65; no prior rehabilitation. |
| Xu 2023 | China | Two-arm randomized controlled clinical study. | Randomized: 58 total; 29/29; Analyzed: 58 total; 29/29. | BFR-LI 60.10±11.22 y; control 59.41±11.85 y. | BFR-LI 19M/10F; control 24M/5F. | BFR-LI hemorrhagic/ischemic 8/21; control 9/20. | Disease duration 1.58±1.28 mo BFR-LI; 1.60±1.27 mo control. | Unilateral stroke with lower-limb dysfunction; BFR-LI left/right 11/18; control 9/20. | Brunnstrom 3.38±0.49 vs 3.45±0.51; age 50–75. |
| Yang 2021 | China | Two-arm randomized clinical study. | Randomized: 66 total; 33/33; Analyzed: 66 total; 33/33. | BFR 46.21±4.98 y; control 46.26±5.21 y. | BFR 15M/18F; control 14M/19F. | BFR hemorrhage/infarction 15/18; control 16/17. | BFR 17.95±3.52 d; control 18.23±3.62 d. | First-ever unilateral stroke with hemiplegia and obesity; MRI/CT-confirmed cerebral hemorrhage or infarction. | BMI ~27.16 kg/m²; Glasgow coma scale >8; age 18–70. |

NR, not reported.

**Table S5B. Design, eligibility, follow-up, and registration characteristics.**

| **Study** | **Randomization / allocation concealment** | **Blinding** | **Setting / recruitment** | **First / recurrent stroke** | **Key eligibility criteria** | **Intervention duration / follow-up** | **Registration / ethics** |
| --- | --- | --- | --- | --- | --- | --- | --- |
| Ahmed 2024 | Randomization: Validated web-based Research Randomizer, 1:1; Allocation concealment: Generated random numbers placed in sealed envelopes; allocation performed by physiotherapist with no study role. | Outcome evaluations performed by a different physiotherapist blinded to allocation (assessor-blinded). | Hospital-based rehabilitation setting; exact recruitment dates were not reported in the article. | First-ever unilateral ischemic stroke. | Inclusion: Ischemic stroke survivors eligible for resistance/aerobic training; detailed criteria reported in paper; Exclusion: Detailed cardiovascular/medical contraindications reported in paper; BFR monitoring included BP/HR/pain. | 5 weeks; resistance training 40 min, 3 d/week; plus aerobic 20 min; Follow-up: No long-term follow-up. | Prospectively registered at ClinicalTrials.gov (NCT05281679); institutional ethics approval (E-74555795-050.01.04-335759). |
| An 2026 | Randomization: Participants were numbered by inclusion order and allocated by random-number table to control, 40%AOP, 50%AOP, or 60%AOP groups. Allocation-concealment procedure was not reported. | Outcome assessor was unaware of group assignment. | Affiliated Hospital of North China University of Science and Technology; August 2024–August 2025. | First/recurrent stroke status was not reported. | Inclusion: Elderly ischemic stroke with frailty meeting study criteria; detailed diagnostic/functional criteria reported; Exclusion: BFRT/exercise contraindications including DVT and severe cardiovascular or respiratory disease; other study-specific exclusions. | 8 weeks; 2 sessions/week; Follow-up: Assessments at baseline, 4 weeks, and 8 weeks; no later follow-up. | North China University of Science and Technology Ethics Committee No. 2024249. |
| Bai 2025 | Randomization: Computer-based stratified randomization generated by a statistician; participants were stratified by sex, and 1RM strata were used to assign the BFR, LL, and HL groups. Allocation-concealment procedure was not reported. | Investigator and participants reported blinded; statistician knew group assignment. | Department of Neurorehabilitation, First People’s Hospital of Lianyungang; 11 Dec 2023–13 Aug 2024. | First stroke attack. | Inclusion: Age 45–75; first subcortical ischemic stroke with hemiparesis 2 weeks–6 months; unilateral supratentorial lesion; lower-extremity strength ≥3; able to complete fNIRS task; Exclusion: MMSE <20; large cortical lesion >1/3 MCA territory; severe carotid stenosis >90% or cerebral stenosis >75%; generalized instability; severe scalp dermatitis. | 4 weeks, 5 d/week; Follow-up: No post-intervention long-term follow-up reported. | ChiCTR2400087378 (retrospectively registered one month after initiation); institutional ethics KY-20231012001-02. |
| Chen 2023 | Randomization: Random-number table. Allocation-concealment procedure was not reported. | Blinding was not reported. | Department of Neurology, First People’s Hospital of Yibin; January 2019–January 2021. | First-ever cerebral infarction. | Inclusion: Age≥60; Chinese 2018 acute ischemic stroke criteria; unilateral new infarct on CT/MRI; first stroke; stable; hemiplegia; Brunnstrom≥III; Ashworth<II; duration<3 months with no worsening in prior month; AWGS sarcopenia; consent; Exclusion: Residual deficits from prior cerebrovascular disease; quadriplegia/no voluntary activity; cognitive/hearing/language/intellectual impairment; venous thrombosis history/coagulation disorder; neuromuscular disease; arthritis/joint injury/severe osteoporosis/fracture; severe systemic/organ disease, infection or malignancy preventing rehabilitation. | 12 weeks; 30 min/session; once daily; 5 d/week; Follow-up: Outcome assessment after 12 weeks; no later follow-up. | Institutional ethics approval and informed consent reported; trial registration was not reported. |
| Du Jinnan 2022 | Randomization: Random-number table; 30 participants per group. Allocation-concealment procedure was not reported. | Blinding was not reported. | Department of Rehabilitation Medicine, Dongguan Kanghua Hospital; July 2020–July 2021. | No prior stroke history according to inclusion criteria. | Inclusion: CT/MRI-confirmed stroke; conscious; stable vital signs; no cognitive impairment; no prior stroke or other comorbid disease as stated; Exclusion: Mental illness/confusion/communication difficulty; other cause of balance dysfunction; venous thrombosis or peripheral arterial disease. | BFR for 6 weeks, 5 d/week; rTMS described as 20 Hz, 20 min/day with a 5-day course. Post-treatment assessment only; no longer-term follow-up reported. | Hospital ethics approval and informed consent reported; trial registration was not reported. |
| Du Yan 2022 | Randomization: Random-number table; 30 participants per group. Allocation-concealment procedure was not reported. | Blinding was not reported. | Neurology inpatient setting; October 2018–October 2020. | First-ever unilateral stroke. | Inclusion: Older stroke patients meeting national diagnostic criteria; hemiplegia; study-specific stability/functional criteria; Exclusion: Contraindications/medical conditions specified by authors, including thrombotic/coagulation concerns. | 8 weeks; 30 min once daily; 5 d/week. Post-treatment assessment only; no longer-term follow-up reported. | Institutional ethics approval and informed consent reported; trial registration was not reported. |
| Feng 2023 | Randomization: Random-number table; random sequence and group labels sealed in envelopes and held by an independent third party; Allocation concealment: Sealed envelopes managed by independent third party until allocation. | Outcome assessor blinded to group allocation. | Second Affiliated Hospital of Chongqing Medical University; September 2021–June 2022. | First/recurrent stroke status was not reported. | Inclusion: 2019 Chinese cerebrovascular criteria, CT/MRI, age 18–80, Mandarin, able to follow one-step command, consent, lower-limb Brunnstrom II–V, some walking ability under supervision; Exclusion: Aphasia/dysphagia or stroke complications precluding testing; other neurologic/psychiatric disease; severe organ dysfunction; BFR contraindication including venous thrombosis, acute disease under treatment, lymphedema therapeutic band. | 20-day trial (~3 weeks); BFR-ET daily, 7 d/week as reported; Follow-up: Assessments pre, day 10, day 20; no later follow-up. | ChiCTR2100050206; ethics approval 2021(657). |
| Feng 2025 | Randomization: Centralized computer-based 1:1 randomization using a random-number table; Allocation concealment: Randomization list kept by a physician not involved in follow-up measurements. | Main investigator and evaluators blinded to allocated treatment during data collection; participants instructed not to reveal treatment. | Department of Rehabilitation Medicine, Second Affiliated Hospital of Chongqing Medical University; September 2021–October 2022. | First/recurrent stroke status was not specified. | Inclusion: Unilateral ischemic/hemorrhagic stroke verified by records; age 18–80; lower-limb Brunnstrom >II; written consent; Exclusion: Other stroke complications preventing testing; neurological/psychiatric disease other than stroke; difficulty understanding procedures; severe organ insufficiency; venous thrombosis; acute disease under treatment; therapeutic band for lymphedema. | 20 sessions over 4 weeks; BFRET 30 min/day; Follow-up: Post-treatment only in main report. | ChiCTR2100050206; Chongqing Medical University ethics approval reported. |
| Gong 2025 | Randomization: Random-number table; 26 participants per group. Allocation-concealment procedure was not reported. | Treatment and outcome-assessor blinding were not reported. | Xiangtan Central Hospital; October 2023–July 2024. | First/recurrent stroke status was not reported. | Inclusion: Stable stroke confirmed by CT/MRI; <6 months; MMSE >25; lower-limb Brunnstrom≥III; MMT≥2; Ashworth<2; standing balance≥1; able to walk; consent; Exclusion: Clinical deterioration/recurrent stroke; severe heart/liver/kidney dysfunction; neurodegenerative/other neurologic disease; DVT on ultrasound; thrombocytopenia/coagulopathy; severe spasticity/ROM restriction; severe language/visual/cognitive impairment. | 4 weeks; twice daily; 6 d/week. Post-treatment assessment only; no longer-term follow-up reported. | Xiangtan Central Hospital ethics approval 2024-05-026. |
| Guo 2025 | Randomization: Computer random-number method; 20/20; Allocation concealment: Not adequately reported; authors explicitly cite allocation concealment as a methodological limitation. | Participant blinding not adequately reported; authors identify blinding as a limitation. | Department of Rehabilitation Medicine; January–June 2024. | First, unilateral stroke. | Inclusion: Chinese stroke diagnostic criteria; CT/MRI hemorrhage/infarction; first unilateral stroke; age 35–77; MMSE≥24; Brunnstrom II–V; basic walking ability Holden≥1; able to follow rehabilitation instructions; Exclusion: Prior nerve block; MMSE <16; thrombocytopenia/coagulation disorder; severe limb injury/fracture/arthritis/amputation; severe organ dysfunction/circulatory failure. | 4 weeks; suspension training 30 min/session, 5 d/week; BFR once daily. Post-treatment assessment only; no longer-term follow-up reported. | Informed consent reported; ethics approval and trial registration were not reported. |
| Ji 2025 | Randomization: Random-number table; 23 participants per group. Allocation-concealment procedure was not reported. | Outcome assessment by a therapist with >3 years’ experience who was unaware of group assignment. | Zhejiang Rehabilitation Medical Center; January–December 2023. | First stroke. | Inclusion: 2019 Chinese cerebrovascular criteria + CT/MRI; first unilateral stroke; 1–3 months; conscious; age 18–75; able to stand/walk; Exclusion: Acute phase; uncontrolled BP >160/100 or dyslipidemia; arrhythmia, heart failure or hemodynamic instability; severe joint pain; lower-limb varicose veins, DVT, severe skin infection/ulcer; Parkinson/Alzheimer or other motor disorders. | 8 weeks; 5 d/week. Post-treatment assessment only; no longer-term follow-up reported. | Institutional ethics approval; hospital project ZKYJ2316. |
| Li 2025 | Randomization: Random-number allocation; 17 participants per group. Allocation-concealment procedure was not reported. | Outcome evaluation by a qualified rehabilitation expert unaware of group assignment. | Department of Rehabilitation Medicine; March 2022–March 2023. | First/recurrent stroke status was not reported. | Inclusion: Stroke diagnostic criteria with CT/MRI confirmation; onset/disease course <6 months; seated balance level 2; informed consent; Exclusion: Significant cognitive deficit/unwillingness; significant cardiovascular disease or uncontrolled hypertension >160/100 mmHg; peripheral arterial disease; lower-extremity venous thrombosis; complex peripheral neuropathy; anticoagulant plus dual-antiplatelet therapy; other severe organ/systemic disease. | 3 weeks; 5 d/week; twice daily. Post-treatment assessment only; no longer-term follow-up reported. | Ethics committee approval and informed consent reported; trial registration was not reported. |
| Luo 2022 | Randomization: Random-number table. Allocation-concealment procedure was not reported. | Blinding was not reported. | Hospital rehabilitation setting; May–December 2021. | First/recurrent stroke status was not reported. | Inclusion: Age 18–75; adequate understanding/language; good compliance; able to tolerate pressurized training; informed consent; Exclusion: Lower-limb DVT; vascular disease; thromboangiitis obliterans; thrombophlebitis; myocardial infarction, heart failure, pulmonary infarction, pulmonary embolism; refusal. | Protocol states 10–14 d per course, once daily; outcomes were assessed at 1, 2 and 3 weeks, so the effective study duration is ambiguously reported; Follow-up: No follow-up beyond treatment period. | Hospital ethics approval and informed consent reported; trial registration was not reported. |
| Qin 2026 | Randomization: Independent research assistant generated a computer-based 1:1 random sequence, stratified by stroke type; Allocation concealment: Sequentially numbered opaque sealed envelopes; treating physiotherapist opened the next envelope after baseline assessment. | Outcome assessors blinded; treating physiotherapist necessarily aware; participants instructed not to discuss training details with assessors. | Rehabilitation Medical Center; July 2020–December 2022. | First-ever stroke or recurrent stroke without significant residual impairment from prior events. | Inclusion: Chinese 2019 cerebrovascular diagnostic criteria; CT/MRI confirmation; first or eligible recurrent stroke; SBP 110–180 mmHg, DBP 60–110 mmHg, HR 50–100 bpm, SpO2≥95% for 24 h; conscious; MMSE >17; consent/cooperation; Exclusion: Severe cognitive dysfunction; severe heart/liver/kidney failure; deaf-mute/comprehension disorder; mental illness/tumor; cerebellar/vestibular balance disorder; treated-limb skin injury; recent/current lower-limb fracture; PVD (ABI<0.9/intermittent claudication/prior PVD intervention); inability to complete training. | 8 weeks, 5 d/week; Follow-up: No long-term follow-up reported after the 8-week intervention. | ChiCTR2500095271; Ethics Committee of the Affiliated Hospital of North Sichuan Medical College, 2023ER378-1. |
| Shang 2025 | Randomization: Computer-generated random numbers; 20 participants per group. Allocation-concealment procedure was not reported. | Intervention personnel could not be blinded; participant and outcome-assessor blinding were not reported. | Daqing Rehabilitation Hospital; September 2023–January 2024. | First, unilateral stroke. | Inclusion: 2019 Chinese cerebrovascular diagnostic criteria; CT/MRI; age 40–70; first unilateral stroke; stable/conscious/able to follow commands; Brunnstrom II–V; Holden≥1; Exclusion: Thrombocytopenia/coagulation disorder; DVT or lymphedema requiring band; MMSE <16; fracture/severe arthritis/amputation; prior botulinum toxin/alcohol nerve block; severe heart/liver/kidney dysfunction or shock. | 4 weeks; 5 d/week. Post-treatment assessment only; no longer-term follow-up reported. | Institutional ethics approval 2023(01). |
| Sun 2024 | Randomization: Random-number table. Allocation-concealment procedure was not reported. | Post-training assessments performed by the same senior therapist who was unaware of group assignment. | Yichang Central People’s Hospital (Xiling campus), Department of Rehabilitation Medicine; October 2021–December 2023. | First stroke. | Inclusion: First stroke confirmed by CT/MRI; stable; <6 months; age 60–85; hemiplegia; standing balance 2–3; walking ≥30 m; consent; Exclusion: Unstable disease; severe cardiopulmonary dysfunction, osteoporosis or joint disease; prior motor/balance disability; severe spasticity; dizziness/cognitive/language/vision/hearing issues; cerebellar/brainstem infarct; BFRT contraindications including DVT/coagulation disorder; refusal/withdrawal. | 6 weeks, 5 d/week; report notes a 2-week interruption within the WBV program; Follow-up: CB&M assessed at 1 and 3 months after discharge. | Ethics review approval reported; trial registration was not reported. |
| Tang 2025 | Randomization: Patients were randomly divided; sequence-generation and allocation-concealment procedures were not reported. | Blinding was not reported. | Rehabilitation Department, First Hospital of Shanxi Medical University. | First stroke. | Inclusion: MRI-confirmed unilateral basal-ganglia infarction; age 30–65; first onset; no prior rehabilitation; duration≤8 weeks; sitting balance2, standing balance≥2; Brunnstrom≥3; MAS≤1+; Exclusion: Severe heart/liver/kidney impairment; coagulation disorder; lower-limb peripheral nerve injury; severe weight-bearing joint OA/other lower-limb dysfunction; vision/hearing/vestibular disorders; severe language/cognitive impairment. | 2 weeks; BFR 5 d/week; Follow-up: Assessment at baseline, 1 week, and 2 weeks. | Ethics approval and trial registration were not reported. |
| Xu 2023 | Randomization: Random-number table; 29 participants per group. Allocation-concealment procedure was not reported. | Blinding was not reported. | Department of Rehabilitation Medicine, Zhongda Hospital, Southeast University. | First/recurrent stroke status was not reported. | Inclusion: Age 50–75; stroke meeting national diagnostic criteria; unilateral limb impairment; study-specific functional criteria; Exclusion: Severe cognitive/consciousness impairment and other contraindications; study excluded thrombotic/coagulation and severe medical conditions as reported. | 2 weeks; twice daily; 6 d/week. Assessments were performed at baseline and 2 weeks; no longer-term follow-up reported. | Ethics approved by Zhongda Hospital, Southeast University; clinical-trial approval/registration No. 2020ZDSYLL277-Y01 as reported. |
| Yang 2021 | Randomization: Participants were randomly divided; sequence-generation and allocation-concealment procedures were not reported. | Blinding was not reported. | Jiaozuo Second People’s Hospital; February 2018–February 2020. | First unilateral stroke. | Inclusion: MRI/CT-confirmed hemorrhagic or ischemic stroke; first unilateral stroke; age 18–70; GCS>8; obesity/hemiplegia population; Exclusion: Other neurologic dysfunction; liver/kidney/heart organ dysfunction; aphasia or blood-function disorder. | 6 weeks. Post-treatment assessment only; no longer-term follow-up reported. | Ethics approval and trial registration were not reported. |

NR, not reported. Studies are presented in alphabetical order by first author surname; studies with the same first author are ordered chronologically by publication year.

**Table S6A. BFRT cuff/device and pressure-prescription characteristics.**

| **Study** | **BFRT arm** | **Device / cuff** | **Cuff size** | **Cuff placement / laterality** | **Pressure prescription basis** | **Target pressure** | **AOP / LOP determination** | **Pressurization / deflation pattern** |
| --- | --- | --- | --- | --- | --- | --- | --- | --- |
| Ahmed 2024 | LIRT-BFR | Blood-pressure cuff/tourniquet; brand was not reported. | Cuff width and length were not reported. | Proximal affected lower limb; Affected lower limb | Fixed absolute pressure range | 150–160 mmHg | Not used; fixed pressure of 150–160 mmHg. | Cuff maintained during exercise sets and 1-min inter-set rest; deflated during 3-min rest between exercises. |
| An 2026 | 40%AOP, 50%AOP, 60%AOP arms | Theratools quantifiable pneumatic pressure cuff; Inflatable pressure cuff | Cuff width and length were not reported. | Upper arm proximal one-third for upper-limb training; lower-limb cuff at thigh inguinal fold; Training involved upper and lower limbs; exact affected-side cuff application not consistently specified | 40%, 50% or 60% AOP | Upper-limb approximate pressures reported: 40%AOP ~80 mmHg, 50%AOP ~120 mmHg, 60%AOP ~160 mmHg; lower-limb target derived from published AOP–thigh circumference relation. | Lower-limb pressure determined using Loenneke relationship table between individual AOP and thigh circumference rather than direct Doppler measurement. Pressure ramped from 20 mmHg in 20-mmHg increments. | Before first session, five pressure-acclimation cycles at 100 mmHg for 20 s with 9 s release; target pressure reached within ≤20 s. |
| Bai 2025 | BFR | B STRONG, USA; Pneumatic cuff | 7 cm width | Proximal affected lower extremity at inguinal crease; Affected lower limb | Fixed 200 mmHg, described as 80% of 250 mmHg chosen from prior healthy-subject work; not individualized AOP. | 200 mmHg | No individualized AOP measurement reported. | Inflated 10 s before session; pressurized during each 3-min pedaling set; deflated at end of set; reinflated during last 10 s of the 1-min inter-set rest. |
| Chen 2023 | BFRT+LIRT | Tourniquet cuff | 7 cm × 50 cm | Upper arm midsection during upper-limb training; femoral midsection during lower-limb training; Paretic-side training implied | ≤1.3 × systolic BP | 140–200 mmHg | No individualized AOP measurement. | Inflated 5 s before each set; deflated after each set; reinflated during last 5 s of inter-set rest. |
| Du Jinnan 2022 | BFR+rTMS | B STRONG BFR band; Inflatable BFR band | B STRONG band with an 18–56 cm circumference range; band width was not reported. | Affected femoral midsection; Affected lower limb | Fixed absolute pressure | 250 mmHg | Not used; fixed pressure of 250 mmHg. | 10 min on/1 min off/10 min on. |
| Du Yan 2022 | BFR+resistance | Tourniquet cuff | 7 cm × 50 cm | Affected femoral midsection; Affected lower limb | ≤1.3 × systolic BP | 140–200 mmHg | No individualized AOP. | Inflate 5 s before each set; completely deflate after each set; reinflate in last 5 s of inter-set rest. |
| Feng 2023 | BFR-ET | B STRONG, Stray Whales LLC, USA; Inflatable BFR band | Cuff width and length were not reported. | Proximal thigh, as close to trunk as possible; Affected lower limb | Fixed absolute pressure range | 160–200 mmHg | Not used; fixed pressure of 200 mmHg. | BFR band inflated and pressure monitored/adjusted during training; exact on/off cycle not specified. |
| Feng 2025 | BFRET | B Strong pressure bands, Stray Whales LLC, USA; Pressure band/cuff | Cuff width and length were not reported. | Proximal thigh; affected-side laterality was not explicitly stated. | Fixed absolute pressure range | 160–200 mmHg | No individualized LOP/AOP measurement reported. | Pressure bands applied before exercise and gradually inflated; detailed on/off cycle not specified in main report. |
| Gong 2025 | BFR cycling + scalp acupuncture | Theratool non-elastic training band; Non-elastic BFR band | 70 cm length × 10 cm width | Affected proximal thigh; upper/mid thigh; Affected lower limb | AOP assessed before training, but treatment used fixed 120 mmHg; authors state pilot 100–240 mmHg corresponded to ~60%AOP. | 120 mmHg | Ultrasound probe at dorsalis pedis artery; minimum pressure at arterial flow occlusion measured three times and averaged. | Intermittent 3 min on/1 min off ×5. |
| Guo 2025 | BFR+suspension | Zhengzhou Jianheng Medical BFR band; Inflatable BFR band | Cuff width and length were not reported. | Reported as on the thigh, approximately 10 cm from the superior hip-bone margin; anatomical wording preserved from source; Affected lower limb implied | Fixed absolute pressure | 200 mmHg | Not used; fixed pressure of 200 mmHg. | 5 min pressurized/1 min released ×5. |
| Ji 2025 | BFR-AT | Theratools (Henan) blood-flow-restriction device; Inflatable cuff | 10 cm width | Root/proximal affected thigh; Affected lower limb | Fixed absolute pressure | 160 mmHg | No individualized AOP. | 10 min pressurized, 5 min deflated rest, 10 min pressurized. |
| Li 2025 | Experimental BFR | Inflatable cuff; brand was not reported. | 10 cm width | Proximal thigh near groin; Lower limb; affected-side wording not fully explicit in English report | Individualized percentage of AOP | 40–80% AOP; control 10% AOP | Cuff pressure increased in steps around systolic pressure; femoral arterial occlusion scanned by ultrasound; pressure at arterial occlusion defined as AOP. Baseline AOP ~180–184 mmHg. | Authors state pressure was maintained between training bouts and released after the final set; wording is awkward but retained as reported. |
| Luo 2022 | Pressurized training + exercise | Compression/pressurization device; device type and brand were not reported. | Cuff width and length were not reported. | Proximal lower limb; laterality was not specified. | Fixed range | 0.01–0.03 MPa (reported units preserved) | Not used; fixed pressure of 0.01–0.03 MPa. | Pressurization/deflation pattern was not reported. |
| Qin 2026 | RR+BFRT | Pneumatic tourniquet cuff; brand was not reported. | 7 cm width; cuff length was not reported. | Proximal portion of affected thigh; Affected lower limb | Fixed absolute pressure; not individualized to AOP | 160–170 mmHg | Not used for prescription. | Cuff applied during BFRT; limb monitored before/after. Methods text describes an initial 10-min pressurized period, 1-min release, then a second 20-min period, while elsewhere total pressurized time is stated as 20 min/session. |
| Shang 2025 | BFR+suspension | T-bfr BFR training band; Inflatable BFR band | Cuff width and length were not reported. | Thigh, 10 cm above superior patellar border; Affected lower limb | Fixed absolute pressure | 200 mmHg | Not used; fixed pressure of 200 mmHg. | 5 min pressurized/1 min released ×5 during suspension training. |
| Sun 2024 | WBV+BFR combined group | Tourniquet cuff; brand was not reported. | 9 cm × 70 cm | Upper thigh near the thigh root; unilateral/bilateral application was not specified. | Relative to systolic blood pressure | ≤1.3 × SBP; approximately 140–200 mmHg | No individualized AOP. | Cuff inflated 5 s before training; 3 min on/1 min off intermittent cycles. |
| Tang 2025 | BFR + routine rehabilitation | Dedicated inflatable pneumatic cuff; brand was not reported. | Cuff width and length were not reported. | Proximal thigh near inguinal crease; Affected lower limb | Fixed absolute pressure | 70 mmHg | Not used; fixed pressure of 70 mmHg. | Cuff pressure was applied during BFR exercise; between-set deflation was not reported. |
| Xu 2023 | BFR-LI | Tourniquet/pressure cuff | Cuff width and length were not reported. | Thigh mid-upper region, 10 cm above superior patellar border; Affected lower limb | Fixed absolute pressure | 200 mmHg | Not used; fixed pressure of 200 mmHg. | Intermittent 3 min on/1 min off ×5. |
| Yang 2021 | BFR+resistance | Pneumatic nylon pressure band + sphygmomanometer; Inflatable nylon cuff | 7 cm × 50 cm | Affected upper arm, thigh, and femoral midsection depending on trained region; Affected side | Fixed site-specific pressures | Upper arm 60–80 mmHg; thigh 80–100 mmHg; femoral midsection 160–170 mmHg | Not used; fixed site-specific pressures. | 10 min on/1 min off ×2–3; pressure may be reduced 5–10 mmHg and release interval lengthened according to intensity/tolerance. |

AOP, arterial occlusion pressure; LOP, limb occlusion pressure; NR, not reported.

**Table S6B. BFRT exercise prescription and treatment dose.**

| **Study** | **Exercise modality** | **Exercise intensity** | **Exercises** | **Sets / repetitions** | **BFRT session duration** | **Frequency** | **Total intervention duration** | **Co-interventions / comparator** | **Supervision / monitoring / release criteria** |
| --- | --- | --- | --- | --- | --- | --- | --- | --- | --- |
| Ahmed 2024 | Low-intensity resistance training + BFR; separate aerobic training without BFR. | 40% 1RM LIRT-BFR; HIRT comparator 80% 1RM. 1RM reassessed weekly. | Knee extension, hip flexion, hip extension, hip abduction, straight-leg raise, leg press/squat sequence as described. | 3 sets × 10 reps for each of six exercises; 1 min between sets, 3 min between exercises. | Resistance session ~40 min; plus 20 min aerobic without BFR. | 3 d/week | 5 weeks | Co-interventions: 20-min aerobic exercise without BFR in both groups; Comparator: High-intensity resistance training at 80%1RM + same aerobic training. | Supervised rehabilitation training; BP, HR, pain and other physical responses recorded; adverse-event logbook; Medical symptoms/abnormal responses triggered cessation; one hypertension-related BFR withdrawal occurred. |
| An 2026 | Elastic-band low-intensity resistance training. | 30% 1RM. | Upper limb: fly/reverse fly; lower limb: seated knee extension and hamstring resistance exercises. | Each movement ~5 s, 19 repetitions; ~15 min/session; 1-min inter-set rest. | ~15 min/session. | 2 sessions/week | 8 weeks | Co-interventions: 30%1RM elastic-band exercise; Comparator: Same 30%1RM resistance training without BFRT. | Outcome assessor blinded; SBP, DBP, resting HR, and MAS monitored; BFRT/exercise contraindications screened. Specific intra-session stop threshold was not reported. |
| Bai 2025 | MOTOmed Viva 2 resistance-mode cycling; cadence 50 rpm. | 30% 1RM in BFR and LL; HL comparator 80% 1RM. | Seated resistance pedaling. | 3-min warm-up; 5 sets × [3 min pedaling + 1 min rest] = 20 min. | 20 min exercise block (15 min cumulative pressurized pedaling). | 5 d/week | 4 weeks | Co-interventions: Conventional medication, physiotherapy and occupational therapy; Comparator: LL: 30%1RM same cycling without BFR; HL: 80%1RM same cycling without BFR. | Supervised seated cycling program; fNIRS monitoring during designated assessment sessions. Specific BFR vital-sign monitoring and stop/release criteria were not reported. |
| Chen 2023 | Low-intensity resistance training with BFRT. | 10–30% 1RM. | Elastic-band upper- and lower-limb resistance exercises; detailed limb movements described in paper. | 10 repetitions/set; 30 s inter-set rest; 20 sets/day. | 30 min/session. | Once daily, 5 d/week | 12 weeks | Co-interventions: Individualized rehabilitation prescription; Comparator: Same low-intensity resistance training without BFR. | Rehabilitation therapist and brain/heart health manager; training performed under monitoring; Observe muscle tone; symptoms such as palpitations, dizziness, dyspnea; HR and BP monitored if symptoms occur; Stop training immediately for increased tone, palpitations, dizziness, dyspnea; rest and call physician as needed. |
| Du Jinnan 2022 | BFRT combined with high-frequency repetitive transcranial stimulation and routine rehabilitation. | Exercise intensity during the pressurized periods was not reported. | BFR pressurization protocol; routine rehabilitation and rTMS were co-interventions. | 10 min pressurized + 1 min release + 10 min pressurized. | 20 min cumulative pressurization. | Once daily, 5 d/week | 6 weeks | Co-interventions: Routine rehabilitation + rTMS (20 Hz; treatment parameters described in paper); Comparator: Routine rehabilitation + same rTMS without BFR. | Clinical rehabilitation setting; DVT and peripheral arterial disease excluded. Specific BFR monitoring and stop/release criteria were not reported. |
| Du Yan 2022 | Low-load resistance training + BFR. | 10–30% 1RM. | Quadriceps knee extension, hamstring knee flexion, elastic-band hip flexion and hip abduction/gluteal exercises. | 10 reps/set; 30 s inter-set rest; 20 sets/day. | 30 min/session. | Once daily, 5 d/week | 8 weeks | Co-interventions: Resistance training; Comparator: Same resistance training without BFR. | Clinical inpatient rehabilitation setting; specific intra-session monitoring and stop/release criteria were not reported. |
| Feng 2023 | BFR combined with progressive functional exercise training. | Exercise difficulty individually adjusted; no single 1RM prescription for functional ET. | Strength, bridge, core, transfers, squats, standing/balance, weight shift, walking, stairs and higher-level tasks according to function. | BFR-ET 20 min/day; ET 30 min/session; brief rests ≤30 s. | 20 min/day. | BFR-ET once/day, 7 d/week as reported | 20 days (~3 weeks) | Co-interventions: Routine rehabilitation + ET 30 min/day in intervention arm; Comparator: Routine rehabilitation + ET 30 min twice/day. | Professionally trained physical therapists; blood pressure, oxygen saturation, cuff pressure/position checked four times; limb appearance and fatigue/numbness monitored. Specific stop threshold was not reported. |
| Feng 2025 | Functional exercise training with BFR. | Each exercise at approximately 10–15 repetition maximum to moderate fatigue; therapist adjusted difficulty. | Active ROM of affected limb; bridge; transfer training; standing balance; overground walking; stairs when possible. | Each exercise according to 10–15RM principle, one set; exact total repetitions individualized. | 30 min/day. | 20 sessions over 4 weeks, weekends excluded | 4 weeks | Co-interventions: Standard rehabilitation context; Comparator: ET using same functional exercise content without BFR; report states 30 min twice/day for control. | Physical therapists delivered exercise; blood pressure, oxygen saturation, cuff pressure, and posture assessed four times; limb color/dimensions and fatigue/numbness continuously monitored. Specific stop threshold was not reported. |
| Gong 2025 | MOTOmed Viva2 cycling under BFR + scalp acupuncture. | 20% 1RM cycling; 1RM reassessed before each session. | Resistance-mode cycling. | 3 min pressurized + 1 min release ×5. | 20-min cycling block. | Twice daily, 6 d/week | 4 weeks | Co-interventions: Scalp acupuncture; low/intermediate-frequency electrical stimulation; new Bobath therapy; Comparator: Scalp acupuncture + 20%1RM cycling without BFR + same basic rehabilitation. | Clinical rehabilitation staff; AOP and BP before first session; HR and SpO2 closely monitored; reduce pressure immediately if discomfort. Additional precautions for hypertension; Discomfort prompted immediate pressure reduction; dropout criteria included complications/adverse events. |
| Guo 2025 | Redcord suspension training + BFR. | Suspension protocol; no 1RM prescription. | Supine pelvic control/strength, side-lying knee/lateral stability, prone knee-control/core exercises. | Suspension: repeated sets as described; BFR 5 min on/1 min off ×5. | ~25 min cumulative pressurization/session. | Once daily; suspension 5 d/week | 4 weeks | Co-interventions: Conventional rehabilitation; suspension training 30 min/session, 5 d/week; Comparator: Conventional rehabilitation + suspension training without BFR. | Clinical rehabilitation staff; BP, oxygen saturation, limb color and temperature; Immediately release band if distal limb becomes dark/purple or numb. |
| Ji 2025 | Cycle-ergometer aerobic training under BFR. | Target HR 65% × (220−age); resistance adjusted to patient comfort. | PRAX-CRU cycle ergometer. | 10 min training + 5 min cuff deflation/rest + 10 min training. | 20 min active training/session. | 5 d/week | 8 weeks | Co-interventions: Routine post-stroke medication and rehabilitation 1.5 h/day, 5 d/week; Comparator: Same aerobic cycle training without BFR. | Supervised rehabilitation; blinded outcome assessor; Continuous HR and SpO2; distal limb circulation observed; Stop immediately if HR >85% × (220−age) or SpO2 <90%. |
| Li 2025 | Cycle ergometer training. | Resistance/intensity not clearly quantified beyond cycling protocol. | Cycle ergometer. | 2 min exercise + 1 min rest; total training block 10 min. | 10 min/session. | Twice daily, 5 d/week | 3 weeks | Co-interventions: Rehabilitation physician instruction/support; warm-up and cool-down; Comparator: Same cycling with 10% AOP cuff pressure to simulate cuff sensation without substantial restriction. | Training performed under rehabilitation physician instruction/support; AOP determined by femoral-artery ultrasound. Specific intra-session safety monitoring and stop/release criteria were not reported. |
| Luo 2022 | Power bicycle/cycle ergometer with pressurized training. | Exercise intensity was not reported. | Power bicycle training plus routine rehabilitation/exercise therapy. | 10–20 min/session; sets and repetitions were not reported. | 10–20 min/session. | Once daily | Protocol states 10–14 d/course, while FMA assessments continue through week 3. | Co-interventions: Routine rehabilitation and exercise therapy; Comparator: Routine rehabilitation + exercise therapy/cycling without pressurization. | Heart rate, blood pressure, and pulse measured before training; major thrombotic, vascular, and cardiopulmonary conditions excluded. Specific intra-session monitoring and stop/release criteria were not reported. |
| Qin 2026 | Low-load lower-limb therapeutic exercise added to standardized routine rehabilitation. | Approximately 20–30% estimated 1RM. | Seated ankle dorsiflexion/plantarflexion, straight-leg raise, knee extension and knee flexion. | 15 repetitions/set; 30 s inter-set rest. Authors describe two pressurized exercise cycles, but the cycle-duration wording is internally inconsistent. | Reported as 20 min pressurized time/session in summary/table; see QC for internal inconsistency. | Once daily; 5 d/week. | 8 weeks | Co-interventions: Standardized 60-min routine rehabilitation: PNF 15 min, Bobath/NDT 15 min, NMES 15 min, functional/task-specific training 15 min, plus acupuncture integrated as described; Comparator: Same standardized routine rehabilitation without BFRT. | Professional rehabilitation staff; Pre/post limb color, sensation and active movement; ask numbness, paresthesia, pain >7/10, discomfort; BP pre/post; Any adverse event to be recorded/reported immediately; symptoms or concerning limb findings prompt clinical action. |
| Shang 2025 | Intermittent BFR used concurrently with Redcord suspension training. | Suspension difficulty individually progressed via position/pads/rope adjustments. | Multi-position suspension exercises for pelvic/knee stability and lower-limb function. | Suspension exercises typically 3 sets; BFR 5 min on/1 min off ×5. | Approximately 25 min cumulative pressurization within suspension session. | Once daily BFR; 5 d/week | 4 weeks | Co-interventions: Routine rehabilitation in morning; suspension/BFR in afternoon; Comparator: Routine rehabilitation + suspension training without BFR. | Clinical rehabilitation staff; BP, oxygen saturation, cuff position, limb color/temperature, fatigue and numbness; Release band if distal limb becomes dark/purple, local/distal numbness occurs, or dorsalis pedis pulse becomes difficult to palpate. |
| Sun 2024 | Intermittent BFR during whole-body vibration (WBV) program. | WBV frequency 12–20 Hz, amplitude 2–4 mm; knee flexion 15–30°. | Side-alternating vertical whole-body vibration standing. | BFR 3 min pressurized/1 min released; daily 15-min BFR session. WBV program table uses 180/60 s cycles ×5. | 15 min/day. | 5 d/week | 6 weeks | Co-interventions: Conventional rehabilitation + Galileo Delta A WBV 9 min/day; study text notes a 2-week interruption within the 6-week program; Comparator: WBV + conventional rehabilitation, or conventional rehabilitation alone. | Therapist-guided and supervised throughout; DVT/coagulation disorders excluded. Specific physiologic monitoring and stop/release criteria were not reported. |
| Tang 2025 | BFR-assisted squat and straight-leg-raise training. | Low-load functional exercise; no 1RM prescription. | Deep squat and straight-leg raise. | 4 sets / 75 total reps using 30-15-15-15; 30–60 s inter-set rest. | BFRT session duration was not reported. | 5 d/week | 2 weeks | Co-interventions: Routine rehabilitation 30 min/session, twice daily, 5 d/week; Comparator: Routine rehabilitation only. | Inpatient rehabilitation setting; specific intra-session physiologic monitoring and stop/release criteria were not reported. |
| Xu 2023 | MOTOmed Viva2 low-intensity resistance cycling. | 20% 1RM. | Cycle ergometer resistance exercise. | 3 min pressurized + 1 min release ×5; cycling 20 min/session. | 20 min/session. | Twice daily, 6 d/week | 2 weeks | Co-interventions: Routine rehabilitation; Comparator: Same routine rehabilitation + 20%1RM MOTOmed cycling without BFR. | Rehabilitation inpatient setting; specific intra-session monitoring and stop/release criteria were not reported. |
| Yang 2021 | Resistance exercise + BFR. | Authors state exercise intensity at ~80% maximal heart rate; resistance comparator used 5-lb Thera-Band. | Upper- and lower-limb resistance movements with elastic band. | Pressure maintained 10 min, released 1 min, repeated 2–3 times. | Approximately 20–30 min cumulative pressurization depending on 2–3 cycles. | 1–2 sessions/day, 5 d/week | 6 weeks | Co-interventions: Resistance training; comparator Thera-Band exercise 30–40 min, 1–2/day, 3–4 d/week; Comparator: Resistance training without BFR. | Family-assisted resistance exercise described; pressure could be reduced by 5–10 mmHg at each site and release time extended according to exercise intensity. Formal supervision and stop threshold were not reported. |

NR, not reported. Studies are presented in alphabetical order by first author surname; studies with the same first author are ordered chronologically by publication year.

**S5. Study-level contribution to quantitative synthesis**

**Table S7. Study-level contribution to the final quantitative synthesis.**

| **Study** | **FMA-LE** | **BBS** | **ADL** | **6MWT** | **10MWT** | **TUG** | **Sit-to-stand** | **Knee extensor PT** | **Cadence** | **Gait speed** | **Any quantitative data extracted** |
| --- | --- | --- | --- | --- | --- | --- | --- | --- | --- | --- | --- |
| Qin 2026 | P | P | S | P | — | — | — | — | — | — | Yes |
| Bai 2025 | P | — | — | — | — | — | — | P | — | — | Yes |
| Li 2025 | P | — | — | — | — | — | R | — | — | — | Yes |
| Feng 2025 | P | — | — | — | — | P | — | — | — | — | Yes |
| Ahmed 2024 | — | — | P | P | — | P | P | — | P | P | Yes |
| An 2026 | P | P | — | — | P | — | — | — | — | — | Yes |
| Sun 2024 | — | P | — | P | — | — | — | — | — | — | Yes |
| Luo 2022 | S | — | S | — | — | — | — | — | — | — | Yes |
| Gong 2025 | — | P | — | — | — | — | — | — | — | — | Yes |
| Chen 2023 | — | — | P | — | — | — | — | — | — | — | Yes |
| Xu 2023 | P | — | S | — | — | — | — | P | — | — | Yes |
| Shang 2025 | P | — | P | — | P | — | — | — | P | P | Yes |
| Guo 2025 | P | P | P | — | P | — | — | — | — | — | Yes |
| Du Yan 2022 | P | P | P | — | — | — | — | — | — | — | Yes |
| Ji 2025 | — | — | — | P | P | — | P | — | — | — | Yes |
| Feng 2023 | S | — | S | — | — | S | — | — | P | — | Yes |
| Tang 2025 | — | P | — | — | — | — | — | — | — | — | Yes |
| Yang 2021 | — | P | — | — | — | — | — | — | — | — | Yes |
| Du Jinnan 2022 | P | — | S | P | — | — | — | — | — | — | Yes |

Status codes: P, included in the final primary pooled analysis; S, quantitative data available but used only in sensitivity or supplementary analyses or otherwise excluded from the primary pooled model; R, reported separately and not pooled in the primary model; —, outcome unavailable or not eligible. All 19 included studies contributed extractable quantitative data.

**S6. GRADE assessment**

**Table S8. GRADE assessment of certainty of evidence.**

| **Outcome** | **Studies (k)** | **Participants** | **Effect measure** | **Effect (95% CI)** | **I² (%)** | **Risk of bias** | **Inconsistency** | **Indirectness** | **Imprecision** | **Publication bias** | **Certainty** |
| --- | --- | --- | --- | --- | --- | --- | --- | --- | --- | --- | --- |
| FMA-LE | 10 | 646 | MD, points | 2.14 (0.74 to 3.54) | 72.9 | Serious | Serious | Not serious | Not serious | Not suspected; Egger/Begg tests nonsignificant and trim-and-fill did not materially alter the estimate | **Low** |
| BBS | 8 | 581 | MD, points | 4.72 (1.62 to 7.82) | 89.8 | Serious | Serious | Not serious | Serious | Not suspected; formal testing not performed because fewer than 10 studies contributed | **Very low** |
| 6MWT | 5 | 271 | MD, m | 8.01 (2.31 to 13.71) | 38.8 | Serious | Not serious | Not serious | Not serious | Not suspected; formal testing not performed because fewer than 10 studies contributed | **Moderate** |
| 10MWT walking speed | 4 | 322 | MD, m/s | 0.14 (0.03 to 0.25) | 67.5 | Serious | Serious | Not serious | Serious | Not suspected; formal testing not performed because fewer than 10 studies contributed | **Very low** |
| ADL | 5 | 241 | SMD | 0.51 (-0.62 to 1.64) | 88.5 | Serious | Serious | Not serious | Serious | Not suspected; formal testing not performed because fewer than 10 studies contributed | **Very low** |
| TUG | 2 | 68 | MD, s; positive values favor BFRT after direction alignment | -1.13 (-21.62 to 19.36) | 0.0 | Not serious | Not serious | Not serious | Very serious | Not suspected; formal testing not performed because fewer than 10 studies contributed | **Low** |
| 5TSTS/FTSTS | 2 | 76 | MD, s; direction aligned | 2.49 (-27.05 to 32.02) | 85.1 | Serious | Serious | Not serious | Serious | Not suspected; formal testing not performed because fewer than 10 studies contributed | **Very low** |
| Knee extensor peak torque | 2 | 98 | MD, Nm | 11.44 (6.56 to 16.32) | 0.0 | Serious | Not serious | Not serious | Serious | Not suspected; formal testing not performed because fewer than 10 studies contributed | **Low** |
| Cadence | 3 | 99 | MD, steps/min | 6.70 (-25.09 to 38.48) | 73.8 | Serious | Serious | Not serious | Serious | Not suspected; formal testing not performed because fewer than 10 studies contributed | **Very low** |
| Gait speed | 2 | 70 | MD, m/s | 0.18 (0.13 to 0.24) | 0.0 | Serious | Not serious | Not serious | Serious | Not suspected; formal testing not performed because fewer than 10 studies contributed | **Low** |

Note. Certainty was assessed using the GRADE framework. Randomized-trial evidence started at high certainty and was downgraded for risk of bias, inconsistency, indirectness, imprecision, or publication bias when warranted. Positive values indicate improvement in favor of BFRT after direction alignment where applicable. Effect estimates and 95% confidence intervals reflect random-effects models using REML and Hartung-Knapp adjustment.

Risk-of-bias downgrading was informed by the outcome-specific RoB 2 judgments for the studies contributing to each pooled outcome.

**GRADE notes**

FMA-LE: Downgraded for risk of bias because most contributing studies had some concerns, and for substantial heterogeneity.

BBS: Downgraded for risk of bias, substantial between-study heterogeneity, and imprecision. Although the pooled 95% CI excluded the null, it spanned effects below and above the approximately 5-point clinically important change reference reported in some early subacute stroke populations, leaving uncertainty regarding the clinical importance of the effect.

6MWT: Downgraded one level for risk of bias; heterogeneity was not substantial and the pooled CI excluded the null.

10MWT: Downgraded for risk of bias, substantial inconsistency, and imprecision. Only four studies contributed to the primary analysis, and the 95% CI for the pooled walking-speed estimate spanned effects below and above the 0.16 m/s clinical-importance reference reported in a specific subacute-stroke population. In addition, the post hoc grey-literature sensitivity analysis attenuated the estimate to 0.09 m/s (95% CI: −0.03 to 0.21), further increasing uncertainty regarding the robustness of the effect.

ADL: Downgraded for risk of bias, substantial heterogeneity, and imprecision because the stricter standard BI/MBI analysis crossed the null.

TUG: Downgraded two levels for very serious imprecision: only two small studies contributed and the CI was very wide and crossed the null.

5TSTS/FTSTS: Downgraded for risk of bias, substantial heterogeneity, and imprecision; only two studies contributed and the CI crossed the null.

Knee extensor peak torque: Downgraded for risk of bias and imprecision because evidence came from only two small studies despite a consistent positive effect.

Cadence: Downgraded for risk of bias, substantial heterogeneity, and imprecision because the CI was wide and crossed the null.

Gait speed: Downgraded for risk of bias and imprecision because only two small studies contributed; heterogeneity was not detected and the pooled CI excluded the null, but the lower confidence limit approached a small-effect threshold.

**S7. Sensitivity and influence analyses**

**Fig. S1. Sensitivity analysis of FMA-LE including Luo 2022** Sensitivity analysis of FMA-LE after re-including Luo 2022. CI, confidence interval; FMA-LE, Fugl-Meyer Assessment of Lower Extremity; MD, mean difference; REML, restricted maximum likelihood.

**Fig. S2. Leave-one-out sensitivity analysis of FMA-LE** Each row represents the pooled FMA-LE effect after omitting one study. The dashed vertical line indicates the pooled effect from the full 11-study model. CI, confidence interval; FMA-LE, Fugl-Meyer Assessment of Lower Extremity; MD, mean difference; REML, restricted maximum likelihood.

**Fig. S3. Influence diagnostics for FMA-LE** Influence diagnostics for the FMA-LE meta-analysis, including externally standardized residuals, Cook's distance, hat values, and DFBETAS.

**Fig. S4. Sensitivity analysis of FMA-LE after excluding Luo 2022 and digitized Li 2025 data** Sensitivity analysis of the revised FMA-LE model after additionally excluding Li 2025, for which FMA-LE values were digitized from the published figure. CI, confidence interval; MD, mean difference; REML, restricted maximum likelihood.

**S8. Exploratory subgroup analyses**

**Fig. S5. Subgroup analysis of FMA-LE by intervention duration after excluding Luo 2022** Exploratory subgroup analysis of FMA-LE by intervention duration after excluding Luo 2022. Individual study effects, subgroup pooled estimates, heterogeneity, and the between-subgroup test are displayed.

**Fig. S6. Subgroup analysis of FMA-LE by BFRT training modality after excluding Luo 2022** Exploratory subgroup analysis of FMA-LE by BFRT training modality after excluding Luo 2022. Individual study effects, subgroup pooled estimates, heterogeneity, and the between-subgroup test are displayed.

**S9. Assessment of small-study effects**

**Table S9. Assessment of small-study effects for FMA-LE.**

| **Analysis** | **Studies (k)** | **Test** | **Statistic** | **P value** | **Adjusted / estimated effect** | **95% CI** |
| --- | --- | --- | --- | --- | --- | --- |
| FMA-LE including Luo 2022 | 11 | Egger regression test (classical weighted regression) | 0.500 | 0.632 | — | — |
| FMA-LE including Luo 2022 | 11 | Egger regression test (mixed-effects meta-regression) | 0.000 | 0.996 | — | — |
| FMA-LE including Luo 2022 | 11 | Begg-Mazumdar rank correlation test | 0.200 | 0.445 | — | — |
| FMA-LE including Luo 2022 | 11 | Trim-and-fill adjusted pooled effect | 2.560 | 0.011 | 3.31 | 0.77 to 5.86 |
| FMA-LE excluding Luo 2022 | 10 | Egger regression test (classical weighted regression) | -0.210 | 0.841 | — | — |
| FMA-LE excluding Luo 2022 | 10 | Egger regression test (mixed-effects meta-regression) | 0.050 | 0.962 | — | — |
| FMA-LE excluding Luo 2022 | 10 | Begg-Mazumdar rank correlation test | 0.022 | 1.000 | — | — |
| FMA-LE excluding Luo 2022 | 10 | Trim-and-fill adjusted pooled effect | 3.490 | 0.007 | 2.14 | 0.74 to 3.54 |

Formal small-study-effect tests were performed for FMA-LE. Analyses including and excluding Luo 2022 are presented separately. These tests should be interpreted cautiously in the presence of between-study heterogeneity and a limited number of studies.

**Fig. S7. Funnel plot for FMA-LE including Luo 2022** Funnel plot for the FMA-LE analysis including Luo 2022. The vertical line represents the pooled random-effects estimate and dashed lines denote the 95% pseudo-confidence limits.

**Fig. S8. Funnel plot for FMA-LE excluding Luo 2022** Funnel plot for the revised primary FMA-LE analysis after excluding Luo 2022. The vertical line represents the pooled random-effects estimate and dashed lines denote the 95% pseudo-confidence limits.

**Table S10. Sensitivity and exploratory subgroup analyses. Panel A. Selected sensitivity analyses.**

| **Outcome** | **Analysis** | **Subgroup / exclusion** | **Studies (k)** | **Effect measure** | **Estimate** | **95% CI** | **I² (%)** | **P value** |
| --- | --- | --- | --- | --- | --- | --- | --- | --- |
| FMA-LE | Revised primary analysis | Excluded Luo 2022 FMA-LE endpoint | 10 | MD | 2.14 | 0.74 to 3.54 | 72.9 | 0.007 |
| FMA-LE | Sensitivity analysis | Included Luo 2022 | 11 | MD | 3.31 | 0.77 to 5.86 | 94.9 | 0.011 |
| FMA-LE | Sensitivity analysis | Excluded Luo 2022 and digitized Li 2025 data | 9 | MD | 2.02 | 0.76 to 3.28 | 75.5 | 0.002 |
| 10MWT walking speed | Sensitivity analysis | Excluded Guo 2025 | 3 | MD, m/s | 0.11 | 0.08 to 0.14 | 0.5 | <0.001 |
| ADL | Primary / stricter analysis | Standard full BI/MBI only | 5 | SMD | 0.51 | -0.62 to 1.64 | 88.5 | 0.280 |
| ADL | Sensitivity analysis | Broader set including non-standard ADL subscores | 7 | SMD | 0.66 | 0.07 to 1.24 | 86.5 | 0.028 |
| Sit-to-stand | Primary / stricter analysis | 5TSTS/FTSTS completion time | 2 | MD, seconds; positive favors BFRT after direction alignment | 2.49 | -27.05 to 32.02 | 85.1 | 0.479 |
| Sit-to-stand | Exploratory analysis | Broad 3-study sit-to-stand synthesis | 3 | SMD; direction aligned | 0.55 | -0.02 to 1.12 | 54.2 | 0.060 |
| Sit-to-stand | Separate study result | Li 2025 30-s sit-to-stand reported separately | 1 | MD, repetitions | 2.80 | -1.14 to 6.74 | NR | NR |

**Table S10. Panel B. Exploratory subgroup analyses.**

| **Outcome** | **Subgroup analysis** | **Subgroup** | **Studies (k)** | **Effect measure** | **Estimate** | **95% CI** | **I² (%)** | **P value** | **P for subgroup difference** |
| --- | --- | --- | --- | --- | --- | --- | --- | --- | --- |
| FMA-LE | BFRT training modality | Cycling / aerobic | 3 | MD | 2.13 | -0.52 to 4.79 | 47.0 | 0.116 | 0.577 |
| FMA-LE | BFRT training modality | Other / multimodal | 3 | MD | 2.50 | -1.14 to 6.15 | 85.5 | 0.178 | 0.577 |
| FMA-LE | BFRT training modality | Resistance-based | 2 | MD | 2.61 | 1.88 to 3.34 | 0.0 | <0.001 | 0.577 |
| FMA-LE | BFRT training modality | Suspension-based | 2 | MD | 0.47 | -0.85 to 1.80 | 0.0 | 0.484 | 0.577 |
| FMA-LE | Intervention duration | <=4 weeks | 6 | MD | 0.73 | -0.26 to 1.73 | 0.0 | 0.148 | 0.011 |
| FMA-LE | Intervention duration | >4 weeks | 4 | MD | 3.22 | 1.81 to 4.64 | 72.2 | <0.001 | 0.011 |
| BBS | BFRT training modality | Non-resistance / multimodal | 5 | MD | 3.79 | 0.16 to 7.42 | 92.6 | 0.041 | 0.279 |
| BBS | BFRT training modality | Resistance-based | 3 | MD | 6.24 | 3.88 to 8.61 | 52.6 | <0.001 | 0.279 |
| BBS | Comparator type | Matched active comparator | 6 | MD | 5.85 | 2.78 to 8.92 | 89.7 | <0.001 | 0.149 |
| BBS | Comparator type | Routine rehabilitation control | 2 | MD | 1.58 | -1.00 to 4.16 | 56.8 | 0.229 | 0.149 |
| BBS | Intervention duration | <=4 weeks | 3 | MD | 5.68 | -0.12 to 11.48 | 92.6 | 0.055 | 0.720 |
| BBS | Intervention duration | >4 weeks | 5 | MD | 4.30 | 1.35 to 7.25 | 87.9 | 0.004 | 0.720 |
| ADL | BFRT training modality | Other / multimodal | 2 | SMD | 0.98 | 0.39 to 1.57 | 63.0 | 0.001 | 0.106 |
| ADL | BFRT training modality | Resistance-based | 3 | SMD | 0.97 | -0.06 to 2.00 | 88.6 | 0.064 | 0.106 |
| ADL | BFRT training modality | Suspension-based | 2 | SMD | -0.19 | -0.63 to 0.25 | 0.0 | 0.396 | 0.106 |
| ADL | Intervention duration | <=4 weeks | 2 | SMD | -0.19 | -0.63 to 0.25 | 0.0 | 0.396 | 0.016 |
| ADL | Intervention duration | >4 weeks | 5 | SMD | 0.99 | 0.42 to 1.56 | 81.0 | <0.001 | 0.016 |
| 10MWT walking speed | Intervention duration | <=4 weeks | 2 | MD, m/s | 0.16 | 0.01 to 0.31 | 79.2 | NR | 0.956 |
| 10MWT walking speed | Intervention duration | >4 weeks | 2 | MD, m/s | 0.14 | 0.04 to 0.24 | 38.4 | NR | 0.956 |

Subgroup analyses were exploratory. P values for subgroup differences refer to the between-subgroup test and should be interpreted cautiously when subgroup sizes are small or unbalanced.

**Table S10. Panel C. Leave-one-out sensitivity analyses.**

| **Outcome** | **Number of omissions** | **Pooled estimate range** | **I² range (%)** | **Omissions with 95% CI excluding null** | **Most heterogeneity-reducing omission** | **Lowest I² (%)** |
| --- | --- | --- | --- | --- | --- | --- |
| 10MWT walking speed | 4 | 0.108 to 0.176 m/s | 0.0 to 65.7 | 4/4 | Ji 2025 | 0.0 |
| 6MWT | 5 | 5.83 to 9.82 | 0.0 to 50.3 | 5/5 | Qin 2026 | 0.0 |
| ADL | 7 | 0.48 to 0.80 | 81.9 to 87.9 | 3/7 | Du Yan 2022 | 81.9 |
| BBS | 8 | 4.18 to 5.46 | 83.5 to 91.7 | 8/8 | Sun 2024 | 83.5 |
| Cadence | 3 | 1.20 to 14.60 | 0.0 to 87.2 | 1/3 | Feng 2023 | 0.0 |
| FMA-LE | 11 | 2.14 to 3.70 | 72.9 to 95.7 | 11/11 | Luo 2022 | 72.9 |
| Sit-to-stand | 3 | 0.27 to 0.78 | 0.0 to 76.3 | 1/3 | Ji 2025 | 0.0 |

Each analysis sequentially omitted one study and refitted the random-effects model. The table summarizes the range of pooled estimates and heterogeneity across all omissions.

**Fig. S9. Leave-one-out sensitivity analysis of BBS** Each row represents the pooled BBS effect after omitting one study. CI, confidence interval; BBS, Berg Balance Scale; MD, mean difference; REML, restricted maximum likelihood.

**Fig. S10. Subgroup analysis of BBS by intervention duration** Exploratory subgroup analysis of BBS by intervention duration. Individual study effects, subgroup pooled estimates, heterogeneity, and the between-subgroup test are displayed.

**Fig. S11. Subgroup analysis of BBS by comparator type** Exploratory subgroup analysis of BBS by comparator type. Individual study effects, subgroup pooled estimates, heterogeneity, and the between-subgroup test are displayed.

**Fig. S12. Subgroup analysis of BBS by BFRT training modality** Exploratory subgroup analysis of BBS by BFRT training modality. Individual study effects, subgroup pooled estimates, heterogeneity, and the between-subgroup test are displayed.

**S10. Additional sensitivity and subgroup analyses**

**Fig. S13. Leave-one-out sensitivity analysis of 10MWT walking speed Each row represents the pooled 10MWT walking-speed effect after omitting one study. Positive values indicate a higher walking speed in favor of BFRT. CI, confidence interval; 10MWT, 10-meter walk test; MD, mean difference; REML, restricted maximum likelihood.**

**Fig. S14. Sensitivity analysis of 10MWT walking speed after excluding Guo 2025 Positive values indicate a higher walking speed in favor of BFRT. CI, confidence interval; MD, mean difference; REML, restricted maximum likelihood.**

**Fig. S15. Subgroup analysis of 10MWT walking speed by intervention duration Exploratory subgroup analysis of 10MWT walking speed by intervention duration. Individual study effects, subgroup pooled estimates, heterogeneity, and the between-subgroup test are displayed. Positive values indicate a higher walking speed in favor of BFRT.**

**S11. Post hoc grey-literature sensitivity analysis of 10MWT walking speed**

**Table S11. Primary and post hoc grey-literature sensitivity analyses of 10MWT walking speed.**

| **Analysis** | **Studies (k)** | **Participants** | **Effect measure** | **Effect (95% CI)** | **I² (%)** | **τ²** | **P value** |
| --- | --- | --- | --- | --- | --- | --- | --- |
| Primary | 4 | 322 | MD, m/s | 0.14 (0.03 to 0.25) | 67.5 | 0.0026 | P = 0.029 |
| Sensitivity A | 6 | 410 | MD, m/s | 0.09 (−0.03 to 0.21) | 94.4 | 0.0097 | P = 0.102 |
| Sensitivity B | 6 | 410 | MD, m/s | 0.09 (−0.03 to 0.21) | 94.4 | 0.0097 | P = 0.102 |

Note. Lan 2024 8-m robustness analysis: MD = 0.09 m/s, 95% CI −0.03 to 0.21, P = 0.104, I² = 94.7%. Sensitivity A and Sensitivity B were identical because neither additional 10MWT result was excluded under the outcome-specific high-risk-of-bias rule.

Fig. S16. Grey-literature sensitivity analysis of 10MWT walking speed Sensitivity A included all eligible independent grey-literature trials. Sensitivity B additionally applied the outcome-specific high-risk-of-bias exclusion rule; the results were identical because neither added 10MWT result was excluded.

Fig. S17. Robustness analysis of 10MWT walking speed treating Lan 2024 as an 8-m timed segment The pooled estimate was MD = 0.09 m/s (95% CI: −0.03 to 0.21, P = 0.104; I² = 94.7%).

**Fig. S18. Leave-one-out sensitivity analysis of 6MWT** Each row represents the pooled 6MWT effect after omitting one study. CI, confidence interval; 6MWT, 6-minute walk test; MD, mean difference; REML, restricted maximum likelihood.

**Fig. S19. Leave-one-out sensitivity analysis of sit-to-stand** Each row represents the pooled sit-to-stand effect after omitting one study. CI, confidence interval; SMD, standardized mean difference; REML, restricted maximum likelihood.

**Fig. S20. Leave-one-out sensitivity analysis of ADL** Each row represents the pooled ADL effect after omitting one study. ADL, activities of daily living; CI, confidence interval; SMD, standardized mean difference; REML, restricted maximum likelihood.

**Fig. S21. Subgroup analysis of ADL by intervention duration** Exploratory subgroup analysis of ADL by intervention duration. Individual study effects, subgroup pooled estimates, heterogeneity, and the between-subgroup test are displayed. ADL, activities of daily living; SMD, standardized mean difference.

**Fig. S22. Subgroup analysis of ADL by BFRT training modality** Exploratory subgroup analysis of ADL by BFRT training modality. Individual study effects, subgroup pooled estimates, heterogeneity, and the between-subgroup test are displayed. ADL, activities of daily living; SMD, standardized mean difference.

**Fig. S23. Leave-one-out sensitivity analysis of cadence** Each row represents the pooled cadence effect after omitting one study. CI, confidence interval; MD, mean difference; REML, restricted maximum likelihood.

**S12. Safety and adverse events**

**Table S12. Safety monitoring and adverse events reported in the included studies.**

| **Study** | **Safety screening / contraindications** | **Monitoring during BFRT** | **Reported adverse events** | **Serious adverse events** | **Thrombotic / cardiovascular events** | **Pain / neurovascular / skin events** | **Safety-related withdrawal / discontinuation** |
| --- | --- | --- | --- | --- | --- | --- | --- |
| Qin 2026 | Excluded PVD (ABI<0.9, intermittent claudication, prior PVD intervention), recent/current fracture, skin injury, severe organ failure and other inability to train; stable BP/HR/SpO2 required. | Before/after each BFRT session: limb color, sensation, active movement; explicit questioning about numbness, paresthesia, pain >7/10 and discomfort; BP measured pre/post. | No serious treatment-related adverse events; no severe numbness, abnormal delayed soreness, BP fluctuation requiring intervention, or DVT-suggestive symptoms. | One fatal heart-failure complication in RR group judged unrelated to treatment; no BFRT-related SAE. | None reported; no DVT-suggestive symptoms; No BP fluctuations requiring intervention in either group; one non-treatment-related fatal heart-failure complication in control. | No severe numbness or delayed-onset muscle soreness beyond typical levels reported; No clinically important skin event reported. | One control withdrawal following fatal unrelated complication; one RR+BFRT lost to follow-up. |
| Bai 2025 | Excluded generalized instability and major vascular/cerebral stenosis; study involved mild-to-moderate ischemic stroke with MMT≥3. | Detailed BFR-specific physiologic safety monitoring not clearly reported in main methods. | No BFR-specific adverse event count reported. Of six non-completers, two reported unbearable muscle soreness/weakness in the HL group, not the BFR group. | None reported as BFR-related. | Thrombotic and cardiovascular events were not reported. | Two unbearable muscle soreness/weakness events occurred in the high-load group; no BFR-specific pain, neurovascular, or skin event reported. | 6/66 did not complete: 1 fNIRS motion artifact, 2 soreness/weakness (HL), 3 lost/dissociated during follow-up. |
| Li 2025 | Excluded uncontrolled hypertension >160/100 mmHg, significant cardiovascular disease, peripheral arterial disease, lower-limb venous thrombosis, anticoagulant + dual antiplatelet treatment and severe systemic disease. | AOP individualized using femoral-artery ultrasound; specific continuous safety-monitoring protocol during sessions was not reported. | No explicit adverse-event results identified in the article. | No serious adverse events reported. | Thrombotic and cardiovascular events were not reported. | Minor cuff-inflation discomfort or transient muscle soreness resolved without intervention; event count and skin events were not reported. | No dropout reported; 34/34 analyzed. |
| Feng 2025 | Excluded venous thrombosis, severe organ insufficiency, acute disease under treatment and lymphedema therapeutic-band use. | BP, SpO2, cuff pressure and posture assessed four times; limb color/dimensions, fatigue and numbness continuously monitored. | No explicit adverse-event count in main report; no BFRET participant discontinued the allocated intervention. | None explicitly reported in BFRET. | No event reported; venous thrombosis screened/excluded; No event reported. | Fatigue and numbness were continuously monitored; event count and skin events were not reported. | BFRET post-treatment n=20 with no intervention discontinuation; ET had one refusal and one hospital discharge. Gait analysis had one fewer participant per group. |
| Ahmed 2024 | Medical eligibility screening before resistance/BFR exercise; cardiovascular responses monitored. | BP, HR, pain and adverse physical responses monitored and logged. | Authors state no detrimental effects were observed during the study overall. | No SAE reported. | Two high-blood-pressure episodes occurred in the HIRT group and were judged unrelated to the treatment protocol; no thrombotic event reported. | One LIRT-BFR participant reported muscular pain that resolved within 12 h; numbness and skin events were not reported. | 2 medical withdrawals: hypertension in LIRT-BFR, cardiac issue in HIRT; 30/32 completed. |
| An 2026 | Exclusion included DVT and severe cardiovascular/respiratory disease; baseline eligibility for older frail ischemic stroke. | SBP, DBP and resting HR measured at baseline, 4 and 8 weeks; muscle tone also tracked. | No explicit adverse-event count. SBP, DBP and resting HR showed no significant group, time or interaction effects. | None reported. | None reported; DVT excluded at baseline; No significant BP/HR changes reported. | Pain, numbness, neurovascular events, and skin events were not reported. | One participant in each of four groups dropped out due to poor cooperation with training; 196/200 analyzed. |
| Sun 2024 | Excluded severe cardiopulmonary disease and BFRT contraindications including lower-limb DVT and coagulation disorders. | Therapist-guided and supervised training; specific BFR vital-sign monitoring thresholds were not reported. | No explicit BFR adverse-event count reported. | No treatment-related SAE explicitly reported. | Thrombotic and cardiovascular events were not reported. | Pain, numbness, neurovascular events, and skin events were not reported. | 10 total attritions described: combined group 1 voluntary withdrawal + 2 incomplete follow-up; WBV 2 incomplete follow-up; control 2 voluntary, 1 disease recurrence, 1 other disease, 1 incomplete follow-up. |
| Luo 2022 | Excluded DVT, vascular disease, thromboangiitis obliterans, thrombophlebitis, myocardial infarction, heart failure, pulmonary infarction and pulmonary embolism. | HR, BP and pulse measured before exercise/pressurized training. | No explicit adverse-event results reported. | Serious adverse events were not reported. | Thrombotic and cardiovascular events were not reported. | Pain, numbness, neurovascular events, and skin events were not reported. | No dropout reported; 40/40 analyzed. |
| Gong 2025 | Excluded lower-limb DVT detected by ultrasound, thrombocytopenia/coagulation disorder, severe organ dysfunction and other conditions limiting safe training. | Before first training: AOP and BP. During training: HR and SpO2 closely monitored; pressure reduced immediately if discomfort. Special precautions for hypertensive patients. | Actual adverse-event counts were not reported. | Serious adverse events were not reported. | Thrombotic and cardiovascular events were not reported. | Discomfort triggered immediate pressure reduction; event count and skin events were not reported. | No actual withdrawal count due to AEs reported. |
| Chen 2023 | Excluded venous thrombosis history/coagulation disorder, severe systemic/organ disease, infection, malignancy and conditions preventing rehabilitation. | Training under rehabilitation therapist/health-manager supervision. If increased tone, palpitations, dizziness or dyspnea occurred, training stopped; HR/BP checked and physician contacted. | All patients completed 12 weeks; no disease aggravation, readmission due to complications or death reported. | No readmission/death reported during training. | No event reported; venous thrombosis/coagulation disorders excluded; No reported event leading to readmission/death; symptom-triggered monitoring protocol present. | Pain, numbness, neurovascular events, and skin events were not reported. | None; 71/71 completed. |
| Xu 2023 | Study-specific exclusions included severe medical/cognitive limitations; thrombotic/coagulation contraindications were considered. | Specific BFR vital-sign and limb-monitoring protocol was not reported. | No explicit adverse-event results reported. | Serious adverse events were not reported. | Thrombotic and cardiovascular events were not reported. | Pain, numbness, neurovascular events, and skin events were not reported. | No dropout reported; 58/58 analyzed. |
| Shang 2025 | Excluded thrombocytopenia/coagulation disorder, DVT/lymphedema band use, severe organ dysfunction/shock and major limb pathology. | BP, oxygen saturation, cuff position, limb color/temperature, fatigue and numbness monitored. | No explicit adverse-event count reported. | None reported. | No thrombotic event reported; cardiovascular events were not reported. | Numbness was monitored; event count and skin events were not reported. | No dropout; 40/40 completed. |
| Guo 2025 | Excluded thrombocytopenia/coagulation disorder, severe limb injury and severe organ dysfunction/circulatory failure. | BP, oxygen saturation, limb color and temperature monitored. | No explicit adverse-event count reported. | Serious adverse events were not reported. | Thrombotic and cardiovascular events were not reported. | Numbness was a cuff-release criterion; event count and skin events were not reported. | No dropout described. |
| Du Yan 2022 | Older stroke population; study-specific exclusions included conditions limiting safe resistance/BFR training, including thrombotic/coagulation concerns. | Specific intra-session safety-monitoring protocol was not reported. | No explicit adverse-event result reported. | Serious adverse events were not reported. | Thrombotic and cardiovascular events were not reported. | Pain, numbness, neurovascular events, and skin events were not reported. | No dropout reported; 60/60 analyzed. |
| Ji 2025 | Excluded uncontrolled BP >160/100 mmHg, arrhythmia, heart failure/hemodynamic instability, lower-limb varicose veins/DVT, skin infection/ulcer and severe joint pain. | Real-time HR and SpO2; distal limb circulation observed during BFR. | BFR-AT group had no adverse reactions during the 8-week treatment. | None reported. | None reported; DVT excluded; No adverse cardiovascular reaction reported. | No adverse reaction reported; No adverse reaction reported. | None reported; 46/46 analyzed. |
| Feng 2023 | Excluded venous thrombosis, acute disease under treatment and lymphedema therapeutic-band use; other major neurologic/organ disease excluded. | BP, oxygen saturation, cuff pressure/location checked four times; limb appearance plus fatigue/numbness closely monitored. | No venous thrombosis, syncope or other adverse event was reported during the BFR intervention in the article’s safety description. | None reported as BFR-related. | None reported; No syncope reported. | No venous thrombosis, syncope, or other adverse event reported; fatigue/numbness monitored; skin events were not separately reported. | 5 attritions were due to disease change or early discharge, not reported as BFR adverse reactions. |
| Tang 2025 | Excluded severe heart/liver/kidney impairment, coagulation disorder, lower-limb peripheral nerve injury, major joint disease, sensory/vestibular and severe cognitive/language disorders. | Routine clinical observation; specific vital-sign thresholds were not reported. | Two participants developed small groin-area skin ecchymoses/bruises; resolved spontaneously in 2–3 days. No dizziness, palpitations, marked BP elevation or delayed-onset muscle soreness reported. | None reported. | None reported; No dizziness/palpitations/marked BP elevation reported. | No delayed muscle soreness reported; 2 minor groin-area ecchymoses/bruises, self-resolved in 2–3 days. | No dropout reported; 32/32 analyzed. |
| Yang 2021 | Excluded other neurologic dysfunction, organ dysfunction, aphasia and blood-function disorder. | Formal BFR safety monitoring not reported. | No explicit adverse-event results reported. | Serious adverse events were not reported. | Thrombotic and cardiovascular events were not reported. | Pain, numbness, neurovascular events, and skin events were not reported. | No dropout reported; 66/66 analyzed. |
| Du Jinnan 2022 | Excluded venous thrombosis and peripheral arterial disease; other neurologic/communication and balance-confounding disorders excluded. | Specific BFR safety-monitoring protocol was not reported. | No explicit adverse-event results reported. | Serious adverse events were not reported. | Thrombotic and cardiovascular events were not reported. | Pain, numbness, neurovascular events, and skin events were not reported. | No dropout reported; 60/60 analyzed. |

NR, not reported. Absence of an explicit adverse-event count is reported as NR and is not interpreted as zero events.
